# Retinal gene therapy for Stargardt disease with dual AAV intein vectors is both safe and effective in large animal models

**DOI:** 10.1126/sciadv.adt9354

**Published:** 2025-03-26

**Authors:** Rita Ferla, Eugenio Pugni, Mariangela Lupo, Paola Tiberi, Federica Fioretto, Andrea Perota, Roberto Duchi, Irina Lagutina, Carlo Gesualdo, Settimio Rossi, Domenico Ventrella, Alberto Elmi, Benjamin McClinton, Carmel Toomes, Tongzhou Xu, Robert S. Molday, Enrico M. Surace, Francesca Simonelli, Maria L. Bacci, Cesare Galli, Muhammad A. Memon, Naveed Shams, Alberto Auricchio, Ivana Trapani

**Affiliations:** ^1^ AAVantgarde Bio srl, Milan, Italy.; ^2^ Telethon Institute of Genetics and Medicine (TIGEM), Pozzuoli, Italy.; ^3^ Department of Advanced Biomedical Sciences, Federico II University, Naples, Italy.; ^4^ Avantea, Laboratory of Reproductive Technologies, Cremona, Italy.; ^5^ Eye Clinic, Multidisciplinary Department of Medical, Surgical and Dental Sciences, University of Campania Luigi Vanvitelli, Naples, Italy.; ^6^ Department of Veterinary Medical Sciences, University of Bologna, Ozzano dell’Emilia, Italy.; ^7^ Department of Veterinary Sciences, University of Pisa, Pisa, Italy.; ^8^ Leeds Institute of Medical Research, University of Leeds, Leeds, UK.; ^9^ Department of Biochemistry & Molecular Biology, Department of Ophthalmology & Visual Sciences, Centre for Macular Research University of British Columbia, Vancouver, B.C., Canada.; ^10^ Department of Translational Medicine, Federico II University, Naples, Italy.

## Abstract

Retinal gene therapy using dual adeno-associated viral (AAV) intein vectors can be applied to genetic forms of blindness caused by mutations in genes with coding sequences that exceed single AAV cargo capacity, such as Stargardt disease (STGD1), the most common inherited macular dystrophy. In view of clinical translation of dual AAV intein vectors, here we set to evaluate both the efficiency and safety of their subretinal administration in relevant large animal models. Accordingly, we have developed the first pig model of STGD1, which we found to accumulate lipofuscin similarly to patients. This accumulation is significantly reduced upon subretinal administration of dual AAV intein vectors whose safety and pharmacodynamics we then tested in nonhuman primates, which showed modest and reversible inflammation as well as high levels of photoreceptor transduction. This bodes well for further clinical translation of dual AAV intein vectors in patients with STGD1 as well as for other blinding diseases that require the delivery of large genes.

## Teaser

AAV-intein-based gene therapy for Stargardt disease proves to be safe and efficacious in large animal models.

## INTRODUCTION

Stargardt disease type 1 (STGD1) is the most common form of inherited macular dystrophy in humans (*1*). STGD1 is inherited as autosomal recessive and is due to biallelic mutations in *ABCA4*, which encodes a retinal-specific all-trans-retinal transporter located in both rod and cone photoreceptor outer segments where it translocates retinoids from the disc lumen to the cytoplasm. ABCA4 is also present at extremely low levels in the retinal pigment epithelium (RPE) where it has been proposed to enhance endolysosomal function (*2*, *3*). In the absence of functional ABCA4, retinoids generate bisretinoid adducts in the photoreceptors that, during the process of disc shedding and phagocytosis, are lastly deposited in RPE cells. This results in abnormally high levels of lipofuscin pigments, including A2E and all-trans-retinal dimer-phosphatidylethanolamine, which trigger RPE cell death and secondary photoreceptor degeneration (*1*). Increased fundus autofluorescence, as a result of lipofuscin accumulation within the RPE cell layer, is a main hallmark of STGD1. In addition, patients with STGD1 experience a loss of central vision with variable reduction in visual acuity due to progressive bilateral atrophy of the cone photoreceptor-dominated macular region.

No therapeutic options are currently available for STGD1. Gene therapy with adeno-associated viral (AAV) vectors is being widely explored for treatment of inherited retinal diseases given their ability to efficiently transduce photoreceptors and their good safety and efficacy profiles in humans (*4*). However, *ABCA4* delivery by AAV vectors has been hampered by the large size of the *ABCA4* coding sequence [6822 base pairs (bp)], which largely exceeds the AAV packaging capacity of about 5 kb. To overcome this limitation, we have recently developed a dual AAV gene therapy approach, which we named “AAV-*ABCA4*-intein” vectors (*5*). In this approach, each AAV vector encodes for one of the two halves of the ABCA4 protein flanked by short split inteins. Upon subretinal AAV administration, split inteins catalyze the seamless joining of the two halves of the ABCA4 protein, thus reconstituting a functional full-length ABCA4 protein in photoreceptors, at levels that largely exceed those achieved via AAV genome recombination by alternative dual AAV-based strategies (*5*), resulting in therapeutic efficacy in *Abca4^−/−^* mice (*5*).

On the basis of these findings, here we set to evaluate both the therapeutic potential and the safety of subretinal administration of AAV-*ABCA4*-intein vectors in large animal models, which are more relevant toward clinical translation. To this aim, we have developed a large animal model of STGD1 using pigs, which have an eye size similar to humans, comparable rod/cone ratio, and the presence of a cone-rich region adjacent to the optic nerve, the so-called visual streak, which resembles the human macula (*6*). In parallel, we have performed ocular safety and expression studies in wild-type (WT) nonhuman primates (NHPs), which have a proper macula, thus demonstrating that subretinal administration of AAV-*ABCA4*-intein vectors is both effective and safe in the retina of large animal models.

## RESULTS

### Generation and characterization of a STGD1 pig model

**Fig. 1.**
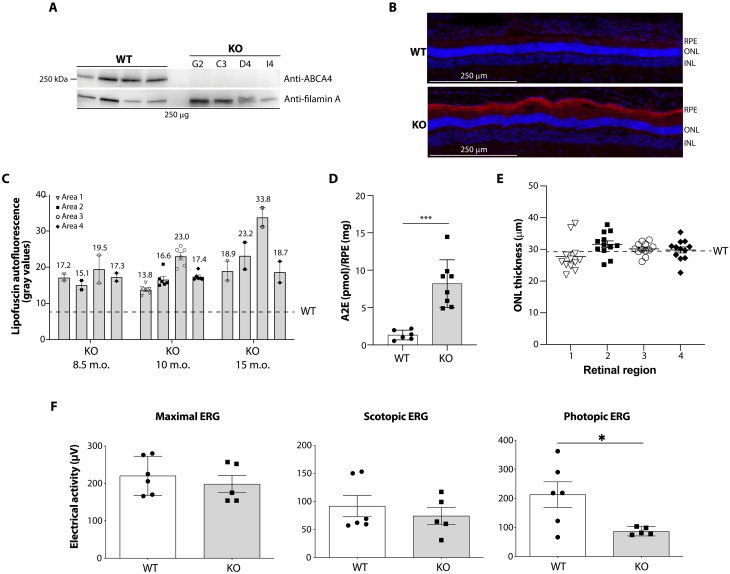
Characterization of the STGD1 pig model. (**A**) Western blot analysis to assess ABCA4 levels in STGD1 (KO) pig retinas derived from different fibroblasts clones. The ID of the clones is indicated above each lane. WT, wild-type. (**B**) Representative images of retinal cryosections from WT and KO pigs at 10 months of age, showing intense lipofuscin accumulation (red autofluorescence in the RPE layer), in the absence of evident retinal degeneration. RPE, retinal pigment epithelium; ONL, outer nuclear layer; INL, inner nuclear layer. (**C**) Quantification of lipofuscin accumulation in KO pig retinas via fluorescence microscopy. Lipofuscin autofluorescence was separately evaluated in four regions of the retina, defined according to the known cone density [fig. S3 and (*6*)]; measurements were done in 8.5-month-old (*n* = 2; i.e., 63OD and 65OD), 10-month-old (*n* = 6; i.e., 7OD, 7OS, 14OD, 14OS, 15OD, and 15OS), and 15-month-old (*n* = 2; i.e., 731OD and 731OS) KO eyes; WT: average lipofuscin levels from *n* = 6 eyes (i.e., 707OD, 707OS, 850OD, 850OS, 857OD, and 857OS). m.o., month-old. (**D**) A2E levels in KO pig RPE; measurements were done in 10- to 15-month-old WT (*n* = 6; i.e., 707OD, 707OS, 850OD, 850OS, 857OD, and 857OS) and KO (*n* = 8; 7OD, 7OS, 14OD, 14OS, 15OD, 15OS, 731OD, and 731OS) eyes. ***P < 0.001, Welch’s *t* test. (**E**) Quantification of ONL thickness in the four retinal regions of KO pig eyes. Measurements were done in 8.5-month-old (*n* = 2; i.e., 63OD and 65OD), 10–10.5-month-old (*n* = 8; i.e., 6OD, 7OD, 7OS, 12OD, 14OD, 14OS, 15OD, and 15OS), and 15-month-old (*n* = 2; i.e., 731OD and 731OS) KO eyes; WT: average levels from *n* = 6 eyes (i.e., 707OD, 707OS, 850OD, 850OS, 857OD, and 857OS). (**F**) Measurements of retinal electrical activity via ERG analysis in KO pigs at 2 to 3 years of age. *P < 0.05, Welch’s *t* test. [(C) to (F)] Data are presented as average ± SEM. Each dot represents a retina.

To generate a pig model of STGD1, we performed somatic cell nuclear transfer (SCNT) experiments starting from pig fibroblasts in which we had induced frameshift mutations in the pig *ABCA4* gene through transfection of a single plasmid expressing both a *Streptococcus pyogenes* Cas9 (SpCas9) and a guide RNA (gRNA), which targets *ABCA4* exon 2. Sanger sequencing results of *ABCA4* exon 2 led to the selection of three male (#C3, C4, and E6) and three female (#D4, G2, and I4) colonies, with biallelic frameshift mutations in this gene, for further SCNT. Of a total of 12 piglets that survived birth, 9 were analyzed and euthanized at different ages to evaluate the course of the STGD1 phenotype (Table 1). Genotyping of DNA extracted from pig tissues confirmed the presence of the expected frameshift mutations originally identified in the *ABCA4*-edited fibroblast clones (fig. S1), which result in the absence of the *ABCA4* protein in pig retinas (Fig. 1A). Further genomic characterization via long range Nanopore sequencing across the edited *ABCA4* locus revealed that larger deletions had occurred in D4- and G2-cloned animals (Table 1). Potential off-target editing events at the top 10 sites predicted by the CRISPOR tool (table S1) were evaluated using primary cells generated from pigs belonging to four different clones (#C3, #D4, #G2, and #I4). Polymerase chain reaction (PCR) products spanning these loci were analyzed using either Sanger or Nanopore sequencing, and no unintended editing outside the *ABCA4* locus was detected (fig. S2).

**Table 1.** Details of STGD1 pigs used in the natural history study for phenotypic characterization. PTC, premature termination codons; WT, wild-type; N.A., not applicable (as they are WT); F, female; M, male.

Pig ID	Clone	Sex	Genotype	Predicted outcome at the mRNA/protein level	Age at euthanasia
764	G2	F	c.83_93del/c.67-622_92del	Frameshift resulting in PTC/incorrect splicing	2.5 months
63	C3	M	c.83_93del/c.86_87insC	Frameshift resulting in PTC	8.5 months
65	C3	M	c.83_93del/c.86_87insC	Frameshift resulting in PTC	8.5 months
7	D4	F	c.84_118del/c.76_161-115del	Frameshift resulting in PTC/incorrect splicing	10 months
14	D4	F	c.84_118del/c.76_161-115del	Frameshift resulting in PTC/incorrect splicing	10 months
15	D4	F	c.84_118del/c.76_161-115del	Frameshift resulting in PTC/incorrect splicing	10 months
6	G2	F	c.83_93del/c.67-622_92del	Frameshift resulting in PTC/incorrect splicing	10.5 months
12	I4	F	c.87del/c.86_87del	Frameshift resulting in PTC	10.5 months
731	G2	F	c.83_93del/c.67-622_92del	Frameshift resulting in PTC/incorrect splicing	15 months
850	N.A.	F	WT	N.A.	10 months
857	N.A.	F	WT	N.A.	10 months
707	N.A.	F	WT	N.A.	16 months

We then evaluated both lipofuscin accumulation and thickness of the retinal outer nuclear layer (ONL) in retinal sections. Already at 8.5 months of age, the first time point analyzed, we found increased lipofuscin accumulation in the RPE of STGD1 pig eyes compared to WT (Fig. 1, B and C). This accumulation was found to occur throughout the entire retina and was more intense in the area centralis, which has the highest cone photoreceptor density (Fig. 1C and fig. S3B). High-performance liquid chromatography (HPLC) analysis of RPE extracts from STGD1 pig eyes confirmed significant accumulation of A2E (Fig. 1D), the main component of lipofuscin in patients with STGD1. Despite this, STGD1 pigs did not show any significant thinning of the ONL compared to WT retinas, up to 15 months of age (Fig. 1E). STGD1 pigs of 2-3 years of age underwent in vivo testing to assess the electrical functionality of the retina via electroretinography (ERG). Although no statistically different electrical responses were found in both maximal and scotopic ERG, photopic ERG, mainly representing the electrical activity of cones, was found to be significantly reduced in STGD1 compared to WT pigs (Fig. 1F).

### Dual AAV intein gene therapy is effective in STGD1 pigs

**Fig. 2.**
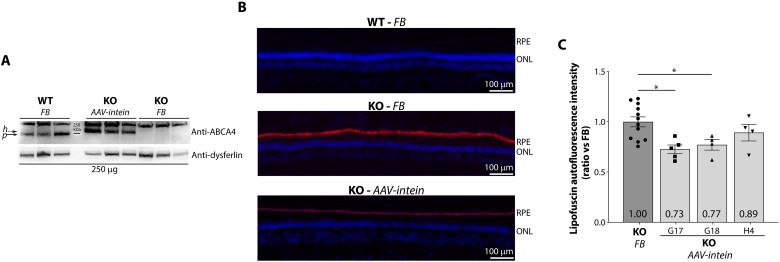
AAV8.A*BCA4*.intein mediated gene therapy in STGD1 pigs. (**A**) Western blot (WB) analysis on retinal lysates from both FB-injected WT eyes and from STGD1 pig (KO) eyes either injected with AAV8.*ABCA4*.intein vectors or with FB. Further details on the areas of the retina used for the WB are provided in fig. S4. The anti-ABCA4 antibody used in the WB reacts with both human (expressed from AAV8.*ABCA4*.intein vectors; dashed arrow) and swine (i.e., endogenous in WT; straight arrow) ABCA4. (**B**) Representative images of retinal cryosections from FB-treated WT (top), FB-treated KO (middle), and AAV8.*ABCA4*.intein injected KO (bottom) eyes. RPE, retinal pigment epithelium; ONL, outer nuclear layer. (**C**) Quantification of lipofuscin accumulation in the area with highest levels of transduction in AAV8.*ABCA4*.intein-treated eyes (2 months postinjection), expressed as ratio relative to the same area in contralateral FB-treated eyes. Light gray bars represent average values (± SEM) of measurements taken in the three AAV8.*ABCA4*.intein-treated KO eyes (pig ID is reported below the graph). The dark gray bar represents average values (± SEM) of measurements taken in the three contralateral FB-treated KO eyes. Dots represent single measurements in each retinal section. Data were analyzed using one-way ANOVA followed by Dunnett’s test, *P < 0.05.

The development of a STGD1 pig model provided the unique opportunity to investigate the therapeutic efficacy of AAV-*ABCA4*-intein vectors in a relevant disease model using the same subretinal injection procedure and similar vector doses to those potentially used in patients. We injected subretinally one of the eyes of two 2-year-old and one 3-year-old STGD1 pigs (i.e., either the left or the right) with a mixture of AAV8.GRK1-*ABCA4*.intein vectors [1 × 10^11^ genome copies (GC) of each vector per bleb, generating two adjacent blebs per eye in the area centralis, which includes the cone-rich visual streak of the retina] and an AAV8.GRK1-*EGFP* vector at a 10-fold lower dose (1 × 10^10^ GC per bleb) to track the transduced area (Table 2 and fig. S4). The contralateral eye in each pig, as well as eyes of WT age-matched pigs (Table 2), were similarly injected with the formulation buffer (FB) used for AAV8.*ABCA4*.intein vectors and a low dose (LD) of AAV8.GRK1-*EGFP* vector (1 × 10^10^ GC per bleb) as control. Naïve, uninjected control eyes were not included as we aimed to use controls accounting for both the potential damage induced by the subretinal injection and the increase in lipofuscin accumulation, which we have previously shown in mice to be induced by the subretinal procedure per se (*7*). Two months postinjection, we harvested the eyes and, based on identification of the enhanced green fluorescent protein (EGFP)–positive areas under a fluorescence stereomicroscope, dissected the eyes into two halves, which were used either for collection of the transduced retina for molecular analysis or for embedding in optimal cutting temperature compound for histological analysis (fig. S4). Western blot analysis in all three AAV8.*ABCA4*.intein injected eyes showed expression of the ABCA4 protein, with a band intensity similar to that of WT pig eyes, which was otherwise absent in the eyes injected with FB alone (Fig. 2A). To define whether the level of ABCA4 reconstitution we achieved in the pig retinas is therapeutically relevant, lipofuscin accumulation in the RPE was analyzed in the retinal regions transduced with AAV8.*ABCA4*.intein vectors, identified based on the presence of EGFP+ photoreceptors. Despite an early time point of analysis postinjection (i.e., 2 months) and the extensive lipofuscin accumulation in 2- to 3-year-old STGD1 pig retinas, all treated eyes showed a reduction in lipofuscin accumulation in the treated area, which reached statistical significance in the areas with highest transduction levels in pigs treated at 2 years of age (Fig. 2, B and C).

**Table 2.** Details of STGD1 pigs used in the AAV8.*ABCA4*.intein gene therapy study. Animals were analyzed at 2 months postinjection. All eyes were coinjected with AAV8.*EGFP* vectors at a dose 10-fold lower than AAV8.*ABCA4*.intein vectors; FB, formulation buffer of AAV8.*ABCA4*.intein vectors (i.e., PBS supplemented with 5% glycerol); WT, wild-type; N.A., not applicable as they are WT; F, female; M, male.

Pig ID	Clone	Sex	Age at injection	Genotype	Left eye	Right eye
G18	D4	F	26 months	c.84_118del/c.76_161-115del	AAV8.*ABCA4*.intein	FB
G17	C3	M	27 months	c.83_93del/c.86_87insC	FB	AAV8.*ABCA4*.intein
H4	E6	M	36 months	c.80_87del/c.86_87insC	AAV8.*ABCA4*.intein	FB
G25	N.A.	F	34 months	WT	FB	FB
H5	N.A.	F	42 months	WT	FB	FB

### Dual AAV intein gene therapy is safe and efficient in the NHP retina

To further evaluate the translational potential of AAV-*ABCA4*-intein vectors to patients with STGD1, we performed a 13-week-long toxicokinetic study in NHPs, not compliant with Good Laboratory Practice (but performed according to scientifically sound principles), to assess the ocular safety and expression in the primate eye. To perform this study, we produced a lot of AAV8.*ABCA4*.intein vectors (table S2) whose potency was initially assessed, before being used in NHPs, in *Abca4^−/−^* mice by evaluation of the levels of expression of full-length ABCA4 protein, which we found to match or even exceed those of *Abca4^+/−^* mice (fig. S5).

NHP eyes received a single subretinal injection of AAV8.*ABCA4*.intein vectors at either an LD (1.8 × 10^11^ total GC per eye, *n* = 7) or a high dose (HD; 5.6 × 10^11^ total GC per eye, *n* = 7) or AAV FB (*n* = 8), according to the experimental scheme reported in Table 3, and underwent comprehensive analyses, as reported in table S3.

**Table 3.** Experimental NHP groups and treatments. Age at treatment was 33 to 41 months. GC, genome copies; FB, formulation buffer of AAV8.*ABCA4*.intein vectors (i.e., PBS supplemented with 35 mM NaCl and 0.001% P188).

Group	Subgroup	Treatment (AAV dose, total GC per eye)		Number of females	Euthanized (weeks)
		Left eye	Right eye		
1	1	FB	AAV8.*ABCA4*.intein (1.8 × 10^11^)	3	13
	2*	FB	AAV8.*ABCA4*.intein (1.8 × 10^11^)	1	5
2	1	FB	AAV8.*ABCA4*.intein (5.6 × 10^11^)	3	13
	2*	FB	AAV8.*ABCA4*.intein (5.6 × 10^11^)	1	5
3	2*	AAV8.*ABCA4*.intein (1.8 × 10^11^)	AAV8.*ABCA4*.intein (5.6 × 10^11^)	3	5

*Half of the eyes collected at week 5 were used for protein expression analysis, whereas the other half was used for experimental purposes not reported in this work.

No mortality, adverse clinical observations, body weight, or food consumption changes were noted throughout the study.

**Fig. 3.**
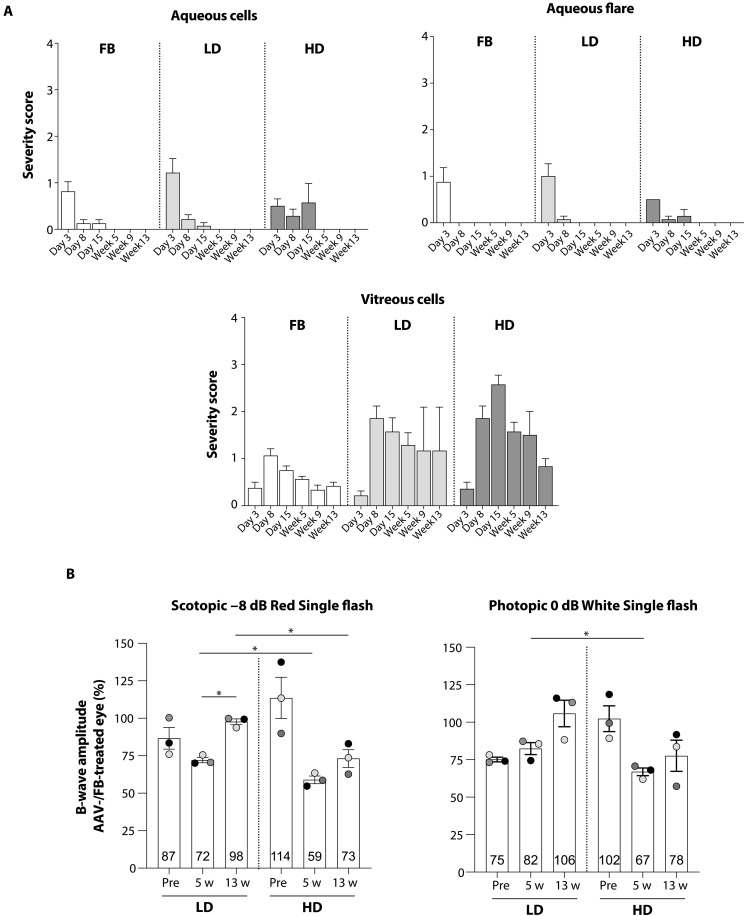
Intraocular inflammation and electroretinography (ERG) responses upon AAV8.*ABCA4*.intein subretinal administration in NHPs. (**A**) Aqueous cells, aqueous flare, and vitreous cells were monitored in injected eyes at different time points postinjection. A score (from 1 to 4) was attributed to define the severity of the ocular findings in each eye. White bars: formulation buffer–treated eyes (FB); light gray bars: eyes treated with a low dose of AAV8.*ABCA4*.intein vectors (LD); dark gray bars: eyes treated with a high dose of AAV8.*ABCA4*.intein vectors (HD). Bars represent average values ± SEM for each group. Detailed number of eyes analyzed and values measured in each eye at each time point are presented in fig. S6. (**B**) ERG values in right eyes treated with either an LD or an HD of AAV8.*ABCA4*.intein vectors are expressed as percentage relative to left FB-treated eyes. Data are presented as means ± SEM. Each dot represents one eye. Pre, analysis performed before injection; w, weeks postinjection. Statistical comparisons were performed using the two-way ANOVA followed by Tukey’s post hoc, *P < 0.05. Further details on statistical analysis, including exact *P* values, can be found in table S7.

Ocular inflammation was monitored by ophthalmic examination (OE), and immunosuppression (IS) was performed in the event of a worsening or nonsubsidence of inflammation, as described in the Materials and Methods section. Overall, the intraocular inflammatory response was initially associated with the surgical procedure and was comparable across all eye groups up to day 8 (Fig. 3A). Minimal (score ≤ 2) aqueous cells and flare were observed on day 3, except in two eyes that showed 3+ aqueous cells (P0001 OD: right eye, LD) and 3+ aqueous flare (P0004 OS: left eye, FB) (fig. S6). Aqueous cells and flare recovered (score ≤ 1) in all eyes on day 8, and in general, anterior inflammation completely disappeared by day 30 in all groups.

As expected after subretinal injections, vitreous cells appeared on day 8 and further increased at day 15 in one of seven LD and four of seven HD eyes, although they were stable or decreased in the other eyes (Fig. 3A and fig. S6). On day 30, the vitreous cell score was in the expected range (≤+2) for this route of administration and improved (7 of 8 FB-treated and 9 of 14 AAV-treated eyes) or remained stable (1 of 8 FB-treated and 4 of 14 AAV-treated eyes) compared to day 15 in all but one LD-treated eye (P0001 OD) (Fig. 3A and fig. S6). A further decrease in vitreous cell score was observed from week 9 to week 13 in all eye groups except for the LD-treated eye of animal P0001 (Fig. 3A and fig. S6).

Signs of posterior inflammation were observed in three HD-treated eyes and recovered following additional IS, except in one animal where they were observed eventually at the end of the study.

Intraocular pressure (IOP) values remained within the expected normal range, except for sporadic (treatment-independent) abnormal lowering (<8 mmHg) observed on day 3 in some eyes with intraocular inflammation (table S4).

To further define potential AAV8.*ABCA4*.intein vector-related adverse effects on NHP retinal and cortical function, we performed both dark-adapted (rod-mediated; Fig. 3B and fig. S7) and light-adapted (cone-mediated; Fig. 3B and fig. S7) ERG, as well as visual evoked potentials (VEPs). Analyses were performed both before injection (predosing) and at 5 and 13 weeks postinjection, using FB-treated contralateral eyes as controls.

A mild and dose-dependent ERG reduction was observed at week 5 in dual AAV8.*ABCA4*.intein-treated eyes. Specifically, only borderline (i.e., mild or at the lower limit of the normal range) amplitude reductions were observed in one of three LD-treated and three of three HD-treated eyes (Fig. 3B and fig. S7). Recovery of dosed-eye ERG amplitudes to values similar to the control eye was largely completed by week 13 in LD-treated eyes and partially completed in HD-treated eyes (Fig. 3B and fig. S7) with one animal (P0102) still presenting borderline reduced ERG. Gray to white retinal to suprachoroidal foci in the injection site were observed at the end of the study in P0102 eye, possibly affecting ERG responses. Last, no alterations were found in VEP measurements.

Fundus photography (FP) and optical coherence tomography (OCT) were performed to provide in vivo insights into potential alterations in either the fundus or retinal layer structure (fig. S8 and table S5). As expected, pigmentary disturbances were seen within the area of the bleb in FB and in AAV8.*ABCA4*.intein-treated eyes, in particular at the injection site. Near-complete resolution of pigmentary disturbance in some animals and partial resolution in others were noted by week 13. Specifically, RPE dappling outside the bleb at week 5 was dose dependent (one of three LD-treated eyes versus three of three HD-treated eyes) and converted in RPE mottling at week 13, indicating a resolution of the pathology (fig. S8).

Accordingly, OCT findings were mainly observed in the injection area, whereas alterations outside the bleb were overall limited (i.e., a focal area of photoreceptor disorganization observed exclusively in one HD-treated eye). A higher incidence of patches of increased hyperreflective material and areas of photoreceptor thinning was observed in AAV8.*ABCA4*.intein-treated eyes compared to controls; patches of moderate retina layers and RPE disorganization were observed exclusively in AAV8.*ABCA4*.intein-treated eyes, whereas areas of ONL thinning were exclusively reported in two of three HD-treated eyes (table S5).

Histopathology was also performed to assess microscopic alterations associated with AAV8.*ABCA4*.intein subretinal administration. At week 5, both LD- and HD-treated NHP eyes showed slight (i) RPE hypertrophy/hyperpigmentation, (ii) outer retina degeneration, and (iii) choroid infiltrates and minimal (iv) photoreceptor nuclei displacement, (v) outer retina, and (vi) vitreous infiltrates (Table 4). At week 13, these findings ameliorated in terms of incidence and/or severity (Table 4). This improvement was more pronounced in the LD-treated eyes, which, overall, were comparable to the FB-injected eyes. Ocular findings were generally focal and restricted to the retina within the dosing site, except for vitreous cell infiltrates, which were diffused, as expected (fig. S9). Microscopic findings were not reported for both brain and optic nerves following histopathology analysis. No AAV8.*ABCA4*.intein-related macroscopic observations or changes in body and brain weights were noted at both interim and terminal sacrifices.

**Table 4.** Incidence and severity of microscopic findings. Incidence and severity of ocular microscopic findings at either interim or terminal euthanasia are reported per each group of treatment. The number (n) of eyes analyzed is reported between parentheses next to each group (FB, LD, HD). Severity scores are defined as follows: minimal, an inconspicuous change; slight, a noticeable but not prominent feature; moderate, a prominent feature; marked, a dominant but not overwhelming feature; severe, an overwhelming condition. Abbreviations: FB, formulation buffer–treated eyes; LD, low dose–treated eyes; HD, high dose–treated eyes. Empty cells correspond to an incidence equal to zero.

	Severity score	Interim euthanasia (incidence)			Terminal euthanasia (incidence)		
		FB (*n* = 1)	LD (*n* = 2)	HD (*n* = 2)	FB (*n* = 6)	LD (*n* = 3)	HD (*n* = 3)
RPE hypertrophy/hyperpigmentation	Slight		1/2	2/2			3/3
	Minimal	1/1			4/6	3/3	
Degeneration outer retina	Slight		2/2	2/2			
	Minimal	1/1					3/3
Displacement of photoreceptor nuclei	Slight						1/3
	Minimal		2/2	1/2		2/3	2/3
Apoptosis of the outer nuclear layer in the fovea	Slight						
	Minimal					1/3	
Degeneration of the inner retina	Slight		1/2				
	Minimal						
Outer retina mononuclear cells infiltrate	Minimal		2/2	2/2			
Perivascular mononuclear cells infiltrate	Minimal			2/2		1/3	1/3
Choroid mononuclear cells infiltrate	Slight		2/2	2/2			1/3
	Minimal	1/1				1/3	2/3
Vitreous mononuclear cells infiltrate	Minimal		2/2	2/2		1/3	1/3

**Fig. 4.**
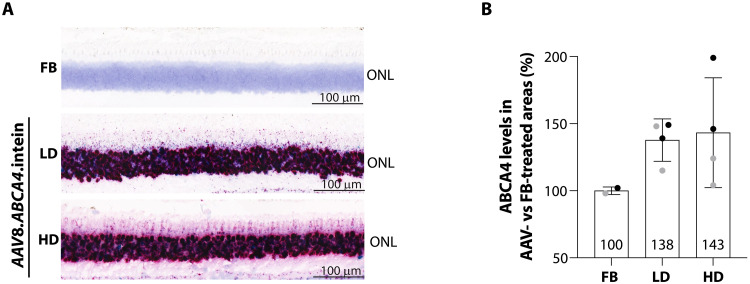
High levels of retinal transduction following subretinal delivery of AAV8.*ABCA4*.intein vectors in NHPs. (**A**) Representative pictures of retinal sections analyzed by BaseScope from NHP eyes at 13 weeks postinjection of either an LD (*n* = 3) or HD (*n* = 3) of AAV8.*ABCA4*.intein vectors or the FB (*n* = 6). Probes annealing to either the *Npu* N-intein (dark green dots) or C-intein (red dots) of human.*ABCA4*.intein mRNAs were used to assess transduction. ONL, outer nuclear layer. (**B**) ABCA4 protein levels were measured by Simple Western analysis in two retina punches [one superior temporal (black dot) and one superior nasal (gray dot) punch] collected from the injected bleb area of eyes receiving either a subretinal injection of AAV8.*ABCA4*.intein at the LD (*n* = 2) or HD (*n* = 2) or FB (*n* = 1) as control. ABCA4 protein levels were normalized to the endogenous control (PDE6B) and then expressed as percentage (%) of ABCA4 protein levels in AAV8.*ABCA4*.intein-treated versus FB-treated areas. Columns represent mean values for group of treatment, whereas dots represent value of each retina punch. Data are reported as means ± SEM.

We then evaluated ABCA4 distribution and levels of expression from AAV8.*ABCA4*.intein vectors in the NHP retina by BaseScope and Simple Western analyses (Fig. 4, A and B).

Retinal sections from the temporal calotte (injection area) of eyes administered with either AAV8.*ABCA4*.intein vectors or the FB, collected at week 13, were analyzed by BaseScope analysis using probes annealing to either the *Nostoc punctiforme* (*Npu*) N-intein or C-intein mRNAs, expressed from vectors 1 and 2, respectively (Fig. 4A). Transduction was found to extend over a large portion of the retinal sections, with the region showing high levels of transduction (76 to 99% of transduced photoreceptors) spanning on average 61% (range: 56 to 69%) of total length of the analyzed retinal sections. Notably, almost total photoreceptor cotransduction was observed (76 to 99% of dual positive photoreceptors). No dose dependency was observed.

To evaluate full-length human ABCA4 protein expression, four 6-mm punches were collected at week 5 from the injected bleb area (one superior temporal punch and one superior nasal punch) and from the not injected area (one inferior temporal punch and one inferior nasal punch) and analyzed by Simple Western analysis. ABCA4 protein levels in AAV8.*ABCA4*.intein-treated retina punches from the injected area were, on average, ~40% above the endogenous ABCA4 levels measured in equivalent samples from the control eye receiving the FB (Fig. 4B). No clear dose dependency was observed as for the BaseScope analysis.

No difference in ABCA4 expression levels were observed between AAV8.*ABCA4*.intein- and FB-injected retina samples from the not injected area (fig. S10). Overall, these data show a good safety and expression profile of AAV8.*ABCA4*.intein vectors in the NHP retina, confirming the promising data from a previous study in mouse models (*5*).

## DISCUSSION

Advancing therapeutic approaches toward clinical testing requires reliable disease models. Although some ex vivo retinal models based on cocultures have been developed recently (*8*), animal models still represent the best option to model inherited retinal diseases. This is particularly true for Stargardt disease (STGD1), where the visual cycle is affected, therefore requiring appropriate models that have intact photoreceptor-RPE interaction. Although exploitation of the *Abca4^−/−^* mouse model provides valuable insights into the efficacy of gene therapies for STGD1, the large differences between the human and mouse eye, including the absence of the primate-specific and cone-rich macular structure in the mouse retina, limit the translatability of the findings. Therefore, in view of clinical translation in patients, in this study we aimed to use as models for testing the therapeutic potential of the AAV-intein platform both the cone-rich pig retina and the NHP retina, which have a proper macula.

We generated an STGD1 pig model by SCNT from Cas9-edited fibroblasts, and we extensively monitored the disease phenotype from 8 to 15 months of age. Although widespread abnormal RPE lipofuscin accumulation was evident from the earliest time point analyzed, no retinal thinning was observed up to 15 months of age. Three of the available animals, which reached 2 to 3 years of age, underwent in vivo ERG analyses before being enrolled in the therapeutic efficacy study. These analyses showed early signs of impaired photoreceptor activity, with a significant reduction in photopic ERG, highlighting that functional retinal deficits and, potentially, retinal degeneration might manifest later in the lifetime of the pig. Our findings are in line with those recently described in a Labrador retriever dog colony found to be homozygous for a frameshift *ABCA4* mutation leading to a premature stop codon in exon 28 (*9*). The canine retinal architecture is similar to the porcine one, with a central horizontal visual streak resembling the human macula. In vivo tests at 10 years of age in this dog model showed abnormalities in both rod and cone electrical activities, although rod function was better preserved. Immunohistochemistry at 12 years of age confirmed significant loss of photoreceptors and increased lipofuscin accumulation in the RPE of the dogs. However, younger dogs were found to manifest only subtle clinical findings (*10*). Therefore, the reduction in photopic ERG at 2 to 3 years of age in STGD1 pigs could precede the additional retinal dysfunctions, as also observed later in life in STGD1 dogs.

We next took advantage of the STGD1 pig model, where the same surgical delivery procedure used in humans can be performed, to test, in a large animal model, the therapeutic efficacy of AAV-*ABCA4*-intein vectors that we have previously shown to be highly effective in *Abca4^−/−^* mice (*5*). In line with those results, in all three ABCA4-KO pig eyes that were injected subretinally with AAV8.*ABCA4*.intein vectors, we found substantial levels of ABCA4 protein reconstitution and a reduction in lipofuscin accumulation, which was statistically significant in the younger animals. Although partial, the reduction in lipofuscin accumulation we observed in eyes treated with AAV8.*ABCA4*.intein vectors appears particularly promising, considering the experimental design that might have limited the extent of rescue, including (i) the availability for this study of ABCA4-KO pigs at an advanced age (i.e., 2 to 3 years), which are expected to have lipofuscin accumulation ongoing already from at least 8.5 months of age (Fig. 1C); and (ii) the combination of the short duration of the study (2 months postinjection), imposed by the complexity of housing Large White pigs, with the use of AAV8, a serotype known to reach a transduction plateau only after 7 weeks (*11*). Whether more prolonged ABCA4 expression mediated by AAV-intein vectors in younger pigs might result in complete normalization of the lipofuscin level remains to be seen. However, the current study suggests that therapeutic benefits can be expected upon AAV-mediated long-term expression of ABCA4 in retinas even at an advanced stage of lipofuscin accumulation, as is expected to occur in patients with STGD1 by the time they are diagnosed.

To further support the therapeutic efficacy of AAV-*ABCA4*-intein vectors in STGD1 pigs, we also attempted to perform both A2E measurements via HPLC and ERG recordings in injected eyes, as performed in STGD1 pig eyes enrolled in the natural history study. However, we could not draw any definitive conclusions due to the limited number of pigs available. ERG measurements were found to present high variability in injected eyes, including those with FB, possibly as a result of the combination of the test variability and of variable degrees of retinal damage upon the injection procedure. Similarly, harvesting of the eyes (fig. S4) relying on the use of a stereomicroscope for dissection of the transduced portion of the retina required storage of the harvested eyes in CO_2_-independent media for 6 to 10 hours, which we found to cause a substantial drop in A2E levels, as assessed by HPLC, even in FB-injected eyes. This highlights one of the limitations of working with large animal models because having a limited number of animals available prevented us from obtaining conclusive data using either of these two tests.

In parallel, in this work we tested both the safety and expression of dual AAV-intein vectors in NHPs, which present the unique features of having the macula, shared only among primates, therefore being a key model to support clinical translation of AAV-intein gene therapy in patients with STGD1. We found subretinal administration of AAV8.*ABCA4*.intein vectors in NHPs to result in mild and dose-dependent ocular structural and functional findings, which largely resolved over time. In addition, ocular findings were generally focal and restricted to the retina within the dosing site, with no macroscopic observations noted during the course of the study. This is in line with what has been observed with other products based on single AAV8 vectors in the NHP and human retina (*12*), suggesting that, under the conditions tested, intein expression does not elicit increased ocular inflammation. Notably, through a BaseScope analysis, we showed that both an LD (1.8 × 10^11^ GC per eye) and an HD (5.6 × 10^11^ GC per eye) of AAV8.*ABCA4*.intein vectors effectively transduces the NHP retina with extremely promising high rates of photoreceptor cotransduction for both vectors across extended retinal regions (e.g., around 60% of the total length of the analyzed retinal sections). Consistently, robust human ABCA4 protein expression was confirmed by Simple Western analysis, which showed, in treated samples, ABCA4 levels ~40% above the endogenous levels measured in control eyes. Given the retinal size and architecture of NHPs, which are highly similar to the human, these results hold promise for further translation of this and other AAV-intein–based approaches, currently under development (*13*, *14*), to the human retina.

In conclusion, in this study we provide evidence of the therapeutic efficacy and safety of dual AAV-intein vectors in large animal models, thus supporting their further clinical translation in patients with STGD1.

## MATERIALS AND METHODS

### Study design

This study was designed to assess both the efficiency and the safety of AAV8.*ABCA4*.intein vectors in the retina of large animal models. To assess the efficiency, we developed a pig model of STGD1, which we characterized by defining development of the main phenotypes associated with STGD1 (i.e., lipofuscin accumulation, retinal degeneration, and abnormalities in the retinal electrical response). In this animal model, we defined therapeutic efficacy by comparing levels of lipofuscin accumulation in AAV.*ABCA4*.intein vector–treated eyes relative to contralateral negative control eyes. In studies evaluating the safety, we subretinally injected NHP eyes and evaluated the appearance of inflammation markers, as well as alterations of the retinal morphology and functionality, as a readout of potential toxicity in AAV.*ABCA4*.intein vector–treated eyes relative to negative control eyes.

In studies involving STGD1 pigs, all available animals were randomly enrolled in studies directed at either characterizing the phenotype or assessing the therapeutic efficacy of AAV.*ABCA4*.intein vectors. In the therapeutic efficacy study, right and left eyes were randomly assigned to each treatment group. Lipofuscin accumulation was quantified objectively using the ImageJ software as detailed in the Materials and Methods section.

Studies involving NHPs were outsourced to external service providers, where expert and/or certified scientific personnel independently performed the listed analysis and provided the data.

Sample sizes in all the presented studies were determined on the basis of previous experience and technical feasibility; the number of biological replicates and measurement for each analysis are indicated in the Results section, figure legends, and Materials and Methods section.

All experimental protocols in ABCA4 KO pigs were approved by the Italian Ministry of Health (Ministero della Salute), as dictated by D.Lgs 26/2014 (nos. 147/2015-PR, 887/2016-PR, and 979/2016-PR).

All procedures in mice were approved from the Italian Ministry of Health (no. 872/2020-PR).

All procedures in NHPs followed the Animal Welfare Act, the Guide for the Care and Use of Laboratory Animals, and the Office of Laboratory Animal Welfare (IACUC/ACUA number: 23-064A).

### ABCA4-KO pig generation via SCNT, maintenance, and euthanasia

ABCA4-KO pigs were generated following the general standard operating procedures (cell culture, transfections, SCNT, recipient sow synchronization, surgical embryo transfer, and postimplantation development) as previously described by Avantea’s research group (*15*, *16*).

Briefly, adult porcine primary fibroblasts (male = 8175; female = 319) were transfected with the pX330-U6-Chimeric_BB-CBh-hSpCas9 plasmid (*17*) (kind gift of F. Zhang; Addgene plasmid no. 42230; http://n2t.net/addgene:42230; RRID: Addgene_42230) encoding for Cas9 and the selected gRNA (gRNA sequence: 5′-gctttgtagtggaactcgtg-3′). Transfected cells were diluted and seeded as single cells in 10 150-mm petri dishes (150 cells per dish). Fifteen days posttransfection, colonies with appropriate morphology and growth rates (∅ ≥ 5 mm) were picked up and cultured for their cryopreservation and for following PCR analyses to assess potential indels in *ABCA4* exon 2 (ABCA4-FW = 5′-ATCCCCCCCCATGACTGAAGTCC-3′; ABCA4-RV = 5′-GCAGATCCAATTCTGGCACCCC-3′; amplicon = 342 bp; LA-Taq, Takara). Purified PCR products (ExoSAP, Thermo Fisher Scientific) were analyzed via Sanger sequencing to select ABCA4-KO colonies, to be used as nuclear donors for further SCNT experiments at AVANTEA (Cremona, Italy).

Young pigs (<150 kg) were housed at the Centro di Biotecnologie A.O.R.N Antonio Cardarelli (Naples, Italy) and maintained under a 12-hour/12-hour light/dark cycle. Large pigs (>150 kg) were housed at the experimental porcine facility of the Department of Veterinary Medical Sciences of the University of Bologna. Animals were housed in single pens, with a 12-hour/12-hour light/dark cycle (minimum of 50 lux during light periods) and a temperature of 22° ± 1°C; pigs were fed a commercial standard diet, with free access to water, and in the presence of dedicated plastic environmental enrichments (Porcichew, Best Balls and Superchallengers; Plexx B.V., ELST, The Netherlands) provided to minimize stress. Animals were daily trained and accustomed, by means of food rewards as positive reinforcement, to interact with humans. From a microbiological point of view, facilities are officially Pseudorabies-free and Swine Vesicular disease-free, and the animals tested negative for Porcine Reproductive and Respiratory Syndrome virus, Porcine Parvovirus and Porcine Circovirus. All procedures were performed according to the Statement for the Use of Animals in Ophthalmic and Vision Research of the Association for Research in Visual Ophthalmology (ARVO) and in compliance with the ARRIVE (Animal Research: Reporting of In Vivo Experiments) guidelines. 

Before either injections or euthanasia, animals were weighed and sedated with an intramuscular injection of a mixture of tiletamine-zolazepam (3 mg/kg, Zoletil; Virbac, Prague, Czech Republic) and dexmedetomidine (0.02 mg/kg, Sedadex; Dechra, Torino, Italy) behind the base of the ear, upon 12 hours of fasting. Once injected, animals were left in a dark and noise-free environment, under strict veterinary monitoring, for 15 min. A peripheral vascular access was achieved through an auricular vein and used to induce general anesthesia by means of an intravenous propofol bolus (2 to 3 mg/kg, Proposure; Merial, France) and to grant fluid therapy (lactated ringer 6 ml/kg per hour) throughout the entire procedure. Immediately after induction, a propofol constant rate infusion (0.1 to 0.2 mg/kg per minute) was started to maintain general anesthesia. Animals were placed in sternal recumbency, orotracheally intubated with a Murphy cuffed tube, and mechanically ventilated (Pressure-controlled ventilation mode set at 12 mmHg, respiratory rate adjusted to maintain normocapnia) upon a single dose of atracurium besylate (1 mg/kg, Tracrium; GSK, Brentford, UK). Temperature was maintained within the physiological range using a Bair Hugger normothermia system (3M, Minnesota, USA).

At specified time points (Tables 1 and 2), animals were euthanized as previously described (*5*), and eyes were harvested and either dissected immediately (for the natural history studies), as detailed in fig. S3, or stored in CO_2_-independent media for 6 to 10 hours and dissected under a steromicroscope (for eyes included in the study evaluating AAV-intein efficiency), as detailed in fig. S4.

### Nanopore sequencing

Nanopore technology was chosen based on its long-range sequencing efficiency to screen genomic large deletions. DNA was extracted from ABCA4-KO pig samples, and the ABCA4 sequence was amplified via long-range PCR using Platinum SuperFi II Green PCR Master Mix (Thermo Fisher Scientific) and 100 ng of DNA as input. Sequencing libraries were prepared using the SQK-LSK109 ligation sequencing kit [Oxford Nanopore Technologies (ONT)]. Bead-based washes were performed using Long Fragment Buffer, and the final library was eluted in 6 μl of Elution Buffer, following a 10-min incubation at room temperature. ONT sequencing was performed using a Flongle Flowcell (R.9.4.1) and MinION instrument. Base calling was performed using Guppy v.5.6.1 (https://nanoporetech.com/). Adaptor sequences were removed using Porechop v.0.2.4 (https://github.com/rrwick/Porechop). Read processing was performed using NanoFilt v.2.8.0 (*18*); the first 75 bases of each read were trimmed, and those reads shorter than 2000 bp and longer than 12,000 bp were removed. Reads with a quality score less than Q10 were also filtered. Sequencing metrics were generated using NanoStat v.1.5.0. Alignment ready reads were mapped to the porcine reference genome (build SusScr11) using MiniMap2 v.2.22 (*19*). Files were manipulated using Samtools v.1.9 (*20*), and aligned reads were visualized using the Integrated Genome Viewer (IGV) v.2.7.2 (*21*). BLAST-like alignment tool (invoked through IGV) was used to obtain primer coordinates and visualize sequence context.

### Off-target evaluation

The top 10 predicted off-target sites were identified using the CRISPOR web tool (CRISPOR.org) with both the NCBI GCF_000003025.6 and Ensembl 76 ensSusScr genome references, sorted by cutting frequency determination (CFD) off-target scores. Off-target sites conserved across both genomes (table S1) were selected for further analysis. Genomic DNA was obtained either from primary fibroblast cell cultures derived from sterile biopsies, as described in (*15*) (for WT animals nos. 8175 and 319 and piglet clones) or from biopsies (in the case of WT animals external to the colony) used as controls. PCR products spanning the selected loci were amplified (details of primers used are in table S6) and initially analyzed by direct Sanger sequencing. For ambiguous results (i.e., PCR with mixed chromatograms suggestive of two different alleles), further analysis was conducted using either Nanopore sequencing (Microsynth) or TOPO-cloning with the Zero blunt vector (Thermo Fisher Scientific), followed by sequencing of at least four colonies per PCR product.

### Western blot analysis

Retinal tissues were lysed in radioimmunoprecipitation assay buffer (supplemented with protease inhibitors and 1 mM phenylmethylsulfonyl fluoride) to extract proteins. After lysis, ABCA4 samples were separated by 6% SDS–polyacrylamide gel electrophoresis, followed by Western blotting. Western blots were blocked with 5% milk and incubated with anti-*ABCA4* antibody (LS-C87292, LifeSpan BioSciences), followed by incubation with anti-dysferlin (MONX10795, MonosanXtra) or anti-filamin (4762S, Cell Signaling) antibodies.

### Histology and fluorescence microscopy for evaluation of lipofuscin accumulation and ONL thickness

Eyes from STGD1 pigs were fixed and sectioned as already described (*5*). Cryosections were mounted with Vectashield with 4′,6-diamidino-2-phenylindole (DAPI) (Vector Lab Inc.) and analyzed under either Leica DM5000 or Zeiss Axioscan microscopes, using appropriate excitation and detection setting for visualization of nuclei (using the DAPI filter), lipofuscin (using TX2 filter, i.e., excitation: 560 ± 40 nm and emission: 645 ± 75) and, in the AAV- or FB-injected eyes, EGFP (using the fluorescein isothiocyanate filter). For each STGD1 pig eye, at least three pictures, in three different sections, were captured in each of the four regions and both analysis of lipofuscin accumulation and measurements of ONL thickness were performed on each acquired image using the NIS-Element software (NIKON). Specifically, for evaluation of lipofuscin accumulation, images were analyzed automatically with NIS-Elements software to obtain a gray value of the red intensity in the RPE layer. A specific threshold ranging between 10 and 25 gray values was set for each STGD1 pig to exclude nonspecific red background. Obtained values were then normalized according to the RPE area measured. A mean value was then calculated for each eye in each of the four measured region. For the assessment of AAV-*ABCA4*-intein efficiency, the area positive for EGFP expression was considered the transduced area.

### HPLC analysis

RPE patches derived from one-quarter of each pig eye were lysed as previously described (*22*) and resuspended in 100 μl of isopropanol. Twenty microliters per sample was analyzed by HPLC as previously described. Measured milli-Absorbance Unit (mAU) values were used to calculate total A2E picomoles in the RPE patches, which were then normalized over the milligram of RPE used for extraction.

### ERG analysis in STGD1 pigs

For electrophysiology recordings, corneal disposable contact lens electrodes (ERG-jet, Universo Plastique, Switzerland) with a drop of benzalkonium chloride polyacrylic acid gel (Lacrinorm, FARMIGEA Holding S.r.l., Pisa, Italy) were used as active electrodes with dermal needle electrodes used as reference placed under the ipsilateral eyelid and aborally on the snout. All electrophysiological data were acquired and amplified using the Retimax system (C.S.O. srl, Florence, Italy). The f-ERG stimuli were produced by a MiniGanzfeld device; 100 sweeps were averaged with a band-pass filter between 1 and 100 Hz, 3k gain, and an acquisition time of 250 ms. Stimuli were flashes of light (3 cd s^−1^ m^−2^) at 1-Hz frequency. Light adaptation was of 20 min (30 cd/m^2^).

### Subretinal injection of AAV-intein vectors in STGD1 pigs

Subretinal injections in pigs were performed as previously described (*23*). Briefly, STGD1 pig eyes were coinjected with two blebs of 100 μl of AAV8 vector solution including AAV8-*ABCA4*-intein (dose of each AAV-intein vector per bleb: 1 × 10^11^ GC) and AAV8.GRK1-*EGFP* (dose: 1 × 10^10^ GC per bleb) vectors. The contralateral eye in each pig and eyes of WT age-matched pigs were similarly injected with the FB used for AAV-intein vectors and an LD of AAV8.GRK1-*EGFP* vector (1 × 10^10^ GC per bleb) as controls. AAV8 vectors were used because of their high transduction efficiency in photoreceptors (*24*). AAV-*ABCA4*-intein vectors were designed and produced as described in (*5*). Briefly, the AAV-*ABCA4*-intein vector mix consists of one vector, which encodes the 5′-half of the human ABCA4 (hABCA4) protein linked at its C terminus to the N-terminal part of the split intein from DnaE of *Npu* (AAV.5′h*ABCA4*.*N-intein*) and a second vector, which encodes the 3′-half of hABCA4 linked at its N terminus with the C-terminal part of the *Npu* DnaE split intein (AAV.C-intein.3′*hABCA4*). Both vectors include the photoreceptor-specific GRK1 promoter (*5*). The two vectors were produced separately and mixed in a 1:1 ratio just before injection.

### Production and characterization of AAV-intein vectors for NHP studies

A single lot of AAV8.*ABCA4*.intein vectors was used in the NHP pilot study. This lot was a 1:1 mixture of the AAV8.5′*hABCA4*.*N-intein* (vector 1) and AAV8.C-intein.3′*hABCA4* vectors (vector 2) (table S2). The two vectors were manufactured by Innovavector srl (Pozzuoli, Italy) through a process based on triple transfection of human embryonic kidney (HEK) 293 cells, followed by two rounds of CsCl_2_ purification. Three sub-lots per vector were produced and pooled together to generate a pooled vector 1 and a pooled vector 2. The physical titer of single and pooled vectors (GC/ml) was determined by TaqMan quantitative PCR (Applied Biosystems, Waltham, MA), using primers and probes designed to anneal to the SV40 polyA. The pooled vector 1 and vector 2 were then mixed in a 1:1 ratio based on the GC titer and filtered through 0.22-μm filters to generate a single lot of AAV8.*ABCA4*.intein vectors. AAV8.*ABCA4*.intein vectors were formulated in an FB composed of phosphate-buffered saline (PBS) (Thermo Fisher Scientific, Waltham, MA) supplemented with 35 mM sodium chloride (Lonza Bioscience, Basel, Switzerland) and 0.001% poloxamer 188 (P188, European Directorate for the Quality of Medicine). Quality control was performed on this lot (table S2) and on the FB used as vehicle control, which were then shipped to the Labcorp (Madison, WI, USA) test facility.

### Subretinal injection of AAV-intein vectors in mice

Pigmented *Abca4^+/−^* and *Abca4^−/−^* mice were generated through successive crosses of albino *Abca4^−/−^* mice (*5*) with Sv129 mice and maintained inbred. Animal were housed at the TIGEM animal facility (Pozzuoli, Naples) and maintained under a 12-hour/12-hour light/dark cycle. Subretinal injections were performed as previously described (*5*), injecting 1 μl of vector mix solution at the indicated dose.

### Test facility, test sites, and animals

Eleven female (range of age 33 to 41 months, not sexually mature) cynomolgus macaques (*Macaca fascicularis*) were used to assess the safety, tolerability, and AAV8.*ABCA4*.intein vectors expression in the retina following subretinal administration. NHPs were housed at LabCorp, under environmental controlled conditions with a 12-hour/12-hour light/dark cycle, which was interrupted exclusively for study-related activities. All procedures followed the Animal Welfare Act, the Guide for the Care and Use of Laboratory Animals, and the Office of Laboratory Animal Welfare.

Cynomolgus macaques received a single subretinal injection per eye according to the scheme reported in Table 3. In vivo and ex vivo safety evaluations were performed at LabCorp in collaboration with OSOD LLC (Madison, WI, USA). mRNA expression analysis by BaseScope was performed by Advanced Cell Diagnostic (Newark, CA, USA), whereas protein expression analysis by Simple Western Size was performed by HD Biosciences Inc. (San Diego, CA, USA).

### Vector formulation and administration

Before administration, AAV8.*ABCA4*.intein vectors were diluted in the FB to reach the target concentration corresponding to the LD and HD; considering that the macaque retinal area is 1.6X smaller than that of humans (*25*, *26*), the LD and HD administered corresponded to 0.3X and 1X, the highest dose currently planned to be used in humans. AAV8.*ABCA4*.intein vectors were administered as a single bleb per eye in the superior retina (180 μl); control eyes were injected with the same volume of FB. Subretinal administration was performed using a DORC subretinal injection device [41-gauge subretinal injection needle, 23 gauge/0.6 mm, ref. 1270 EXT (D.O.R.C. Dutch Ophthalmic Research Center, B.V. Zuidland, The Netherlands)] by a Vitreoretinal Surgeon and assisted by a board-certified veterinary ophthalmologist from LabCorp.

A medication regimen was started 4 days before dosing to minimize surgery-related inflammation and/or infection. Also, a new immunosuppression protocol based on prednisolone was adopted in this study to manage AAV-related inflammation.

### Safety assessments in NHPs

Animals were observed twice daily for mortality, abnormalities, and signs of pain or distress. Detailed observations and body weight measurement were conducted during the predose phase, day 1, and weekly thereafter. Food consumption was recorded daily.

Ocular safety was assessed through (i) in vivo evaluation based on OEs, assessment of full-field ERG parameters, OCT, and FP and (ii) ex vivo through histopathology.

OEs were conducted for all animals once during the predose phase, on days 3, 8, and 15, and once during week 5 and, for subgroup 1 only, on weeks 9 and 13. Animals were anesthetized with ketamine, and pupils were dilated with a mydriatic agent (1% tropicamide) prior to examination using an indirect ophthalmoscope and slit-lamp biomicroscope. A severity score was attributed by a certified veterinary ophthalmologist, as previously described (*27*). IOP measurements were recorded in conjunction with OEs using an applanation tonometer. A topical anesthetic (e.g., 0.5% proparacaine) was applied before IOP measurements.

ERG, OCT, and FP were conducted for all animals once during the predose phase and for subgroup 1 animals once during weeks 5 and 13 of dosing phase. FP was also taken on day 1 in all animals to document bleb formation.

For ERG, animals were anesthetized with ketamine and dexmedetomidine and pupils were dilated with a mydriatic agent. Scotopic, photopic, and VEP tests were performed on each eye. Animals were fasted for at least 2 hours before ERG procedures, dark adapted for at least 1 hour before scotopic tests, and light adapted for at least 10 min before photopic tests. Scotopic tests were conducted using stimuli as follows: (i) a dim short wavelength (Scotopic −34 dB Blue Single Flash) and (ii) a long wavelength (Scotopic −8 dB Red Single Flash); a mixed rod-cone stimulus (Scotopic 0 dB White Single Flash); oscillatory potentials—high-frequency components digitally filtered from the Scotopic 0 dB White Single Flash condition. Photopic tests were conducted using stimuli as follows: (i) single white flashes (Photopic 0 dB Single Flash) and (ii) flashes delivered at a rate of 30.3 Hz (Photopic 0 dB 30.3 Hz White). VEP tests were done using monocular stimulation (with the unstimulated eye occluded); recordings were made unilaterally through each eye. An average of 80 flashes with an interstimulus interval of 0.244 s (4.1 Hz) was used.

For OCT and FP, animals were fasted for at least 10 hours before the procedure and then were anesthetized with ketamine and maintained on sevoflurane. Pupils were dilated with a mydriatic agent for OCT analysis.

Imaging was done in a manner to obtain axial views of the retinal surface in the posterior fundus. The instruments were set to perform standard retinal scans (macular volume scans, line scans, and/or circle scans). A single enhanced-depth imaging volume scan was also taken.

Color photographs of each eye were taken with a digital fundus camera to include stereoscopic photographs of the posterior pole and nonstereoscopic photographs of two midperipheral fields (temporal and nasal).

On the day of euthanasia, animals were anesthetized with sodium pentobarbital, exsanguinated, and necropsied. Terminal body weights were recorded for all animals as well as macroscopic examination of the external features of the carcass, external body orifices, abdominal, thoracic, cranial, pelvic, and oral cavities, organs, and tissues.

Designated tissues, i.e., eyes with bulbar conjunctiva, optic nerve, and brain were collected. Eyes were infiltrated with 4% paraformaldehyde (PFA) in PBS 1X until turgid. Eyes and optic nerves were processed to paraffin block after 24 to 36 hours in 4% PFA. Eyes were trimmed vertically into three parts: nasal calotte, central region (to include optic nerve and optic disc), and the temporal calotte (to include fovea/macula and subretinal bleb). All sections were embedded, sectioned at 5 μm, and stained with hematoxylin and eosin (H&E).

The brain was fixed in 10% neutral buffered formalin and then embedded in paraffin for histopathology and stained with H&E.

### BaseScope

A chromogenic BaseScope HD Duplex ISH assay (catalog no. 323800, Advanced Cell Diagnostic) was performed on sections from the temporal calotte of eyes from groups 1 and 2, subgroup 1 (Table 3). Before testing, RNA quality control was performed by duplex hybridization with positive control probes toward *M. fascicularis* POLR2A (BA-Mfa-POLR2A-3zz, catalog no. 703201) and PPIB (BA-Mfa-PPIB-3zz, catalog no. 703181-C2). Sections were then hybridized with BaseScope custom probes for N-Intein and C-intein portion of human 5′ABCA4.N-Intein and C-Intein.3′ABCA4 mRNAs (catalog nos. 1236621 and 1236631). Horseradish peroxidase- and Alkaline phosphatase-based reactions were used to detect dark green and red signals corresponding to N-Intein and C-Intein mRNA, respectively. DNA removal was performed before hybridization. Imaging was performed on 3D Histech PANNORAMIC SCAN II bright-field scanner. Percentage of positive cells and double-positive cells was scored visually by a qualified scientist based on the number of cells with >1 dot per cell and binned into the following categories: 0, 1 to 25, 26 to 50, 51 to 75, 76 to 99, and 100%. The length of photoreceptor layer was measured for each category using the 3DHistech software.

### Simple Western analysis

Some eyes collected at week 5 (from subgroup 2; Table 3), specifically a single control eye receiving the AAV FB and either LD (*n* = 2) or HD-treated (*n* = 2) eyes, were processed for ABCA4 protein expression analysis, by Simple Western.

The eye posterior calotte was flower petaled on a cold plate to flatten the retina. Four 6-mm punches were collected and maintained separately and included two punches from the bleb area (superior temporal and superior nasal) and two punches from non-bleb areas (inferior temporal and inferior nasal). For each punch, the retina was separated from other components using filter paper and then immediately frozen. Retina sample lysates concentrated to 3 μg/μl (where possible) were denatured by incubation at 37°C for 20 min, using JESS reagents (PS-ST03EZ-8, Biotechne, Minneapolis, MI, USA) according to the manufacturer’s instructions. Separation was performed using 66- to 440-kDa separation module (SM-W0007, Biotechne), and detection was performed using the chemiluminescence channel (High Dynamic Range) by multiplexing the ABCA4 and PDE6B, which is specifically expressed in photoreceptors and thus used as loading control. The following primary antibodies were used: mouse anti-ABCA4 Ab77285 (1:500, Abcam, Cambridge, UK) and rabbit anti-PDE6B PA1-722 (Thermo Fisher Scientific, Waltham, MA, USA). Lysate preparation and Simple Western analysis was performed by HD Biosciences (San Diego, CA, USA). Protein quantification was performed using the Compass for Simple Western (BioTechne) software.

### Statistical analysis

Statistical analysis was performed using GraphPad Prism 10, version 9.2.0. All reported *P* values were obtained from (i) unpaired, two-tailed Student’s (or Welch’s) *t* tests (when comparing two groups) with a confidence level of 95% and *P* < 0.05 considered to be statistically significant or (ii) one-way or two-way analysis of variance (ANOVA) testing, followed by either Dunnett’s or Tukey’s post hoc (when comparing more than two groups) with *P* < 0.05 significance level. Shapiro-Wilk test was used to test for normality of data. Statistical tests for each experiment are indicated in figure legends. Data are presented as means ± SEM. *P < 0.05, **P < 0.01, ***P < 0.001, and ****P < 0.0001. Sample numbers are provided in the figure legends. Exact *P* values of the statistical analysis on data shown in fig. S7 are listed in table S7.
