# Supplementary material for: Retinal gene therapy for Stargardt disease with dual AAV intein vectors is both safe and effective in large animal models
Source: Sci Adv. 2025 Mar 26;11(13):eadt9354. doi: 10.1126/sciadv.adt9354 (PMC11939046; doi:10.1126/sciadv.adt9354)
Supplement: Supplementary file 1 — Figs. S1 to S10 Tables S1 to S7 [file sciadv.adt9354_sm.pdf]

Science Advances
AAAS

Supplementary Materials for

Retinal gene therapy for Stargardt disease with dual AAV intein vectors is both safe and effective in large animal models

Rita Ferla *et al.*

Corresponding author: Ivana Trapani, trapani@tigem.it

Sci. Adv. **11**, eadt9354 (2025)
DOI: 10.1126/sciadv.adt9354

This PDF file includes:

Figs. S1 to S10
Tables S1 to S7

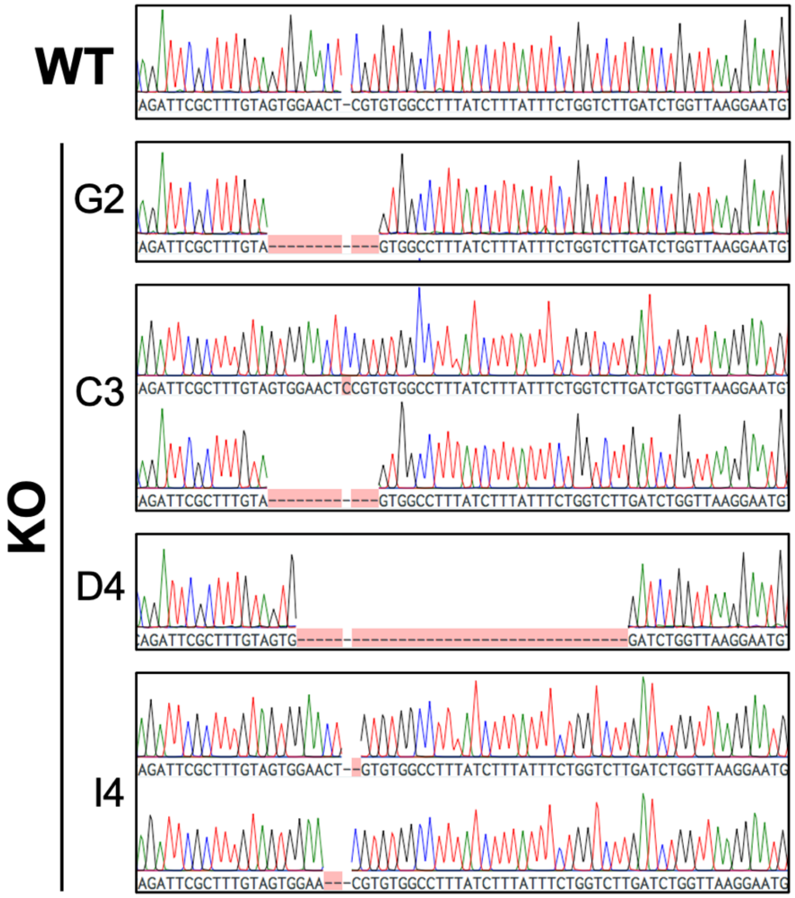

Figure S1. Genotypes of the ABCA4 KO pigs.

Representative chromatograms of Sanger sequencing analysis of DNA extracted from ABCA4 KO pigs.

The ID of the clones is indicated on the left.

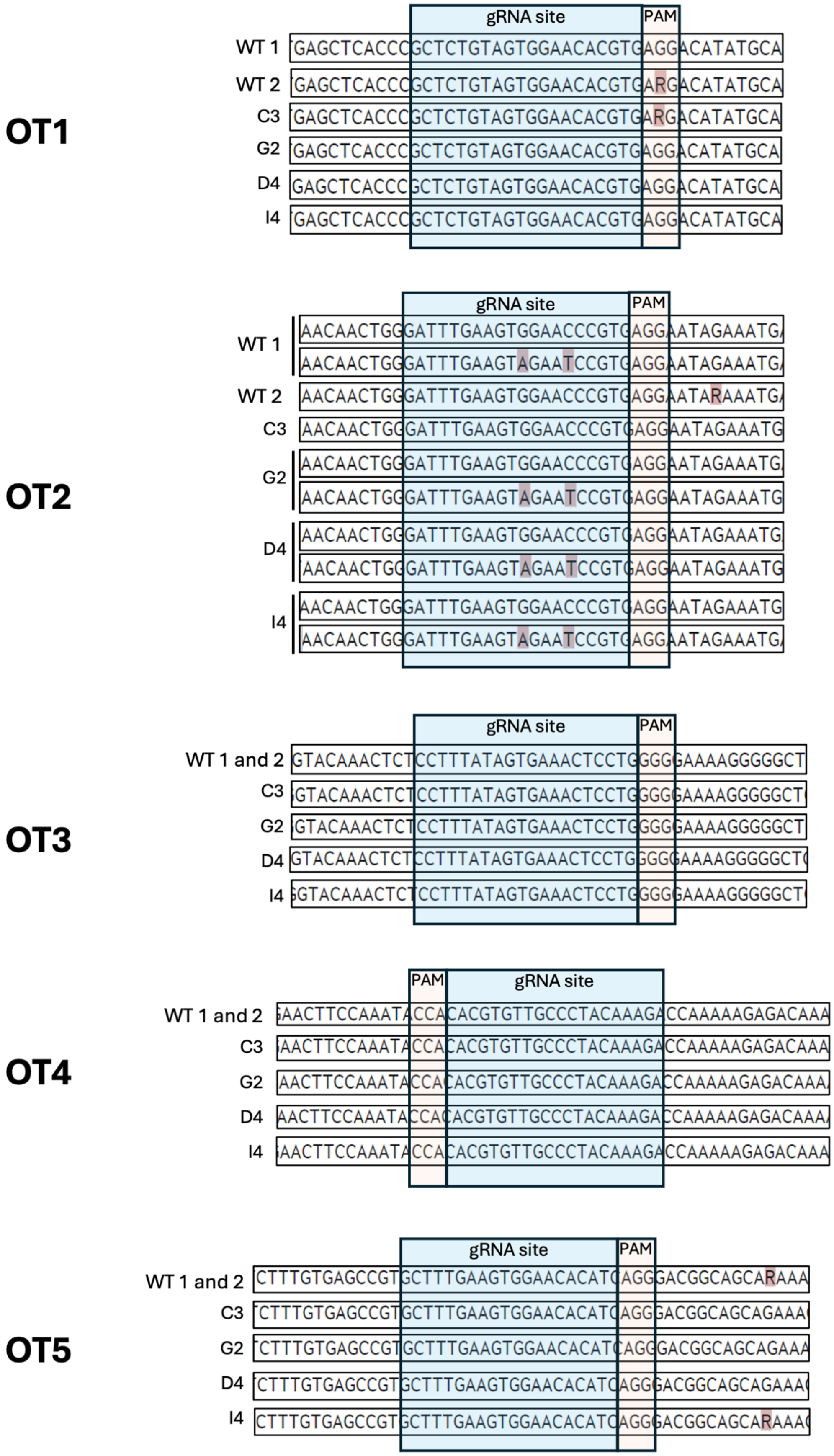
OT1
gRNA site
PAM
WT 1
GAGCTCACCCGCTCTGTAGTGGAACACGTGAGGACATATGCA
WT 2
GAGCTCACCCGCTCTGTAGTGGAACACGTGARGACATATGCA
C3
GAGCTCACCCGCTCTGTAGTGGAACACGTGARGACATATGCA
G2
GAGCTCACCCGCTCTGTAGTGGAACACGTGAGGACATATGCA
D4
GAGCTCACCCGCTCTGTAGTGGAACACGTGAGGACATATGCA
I4
GAGCTCACCCGCTCTGTAGTGGAACACGTGAGGACATATGCA
OT2
gRNA site
PAM
WT 1
AACAACTGGGATTTGAAGTGGAACCCGTGAGGAATAGAAATG
AACAACTGGGATTTGAAGTAGAATCCGTGAGGAATAGAAATG
WT 2
AACAACTGGGATTTGAAGTGGAACCCGTGAGGAATARAAATG
C3
AACAACTGGGATTTGAAGTGGAACCCGTGAGGAATAGAAATG
G2
AACAACTGGGATTTGAAGTGGAACCCGTGAGGAATAGAAATG
AACAACTGGGATTTGAAGTAGAATCCGTGAGGAATAGAAATG
D4
AACAACTGGGATTTGAAGTGGAACCCGTGAGGAATAGAAATG
AACAACTGGGATTTGAAGTAGAATCCGTGAGGAATAGAAATG
I4
AACAACTGGGATTTGAAGTGGAACCCGTGAGGAATAGAAATG
AACAACTGGGATTTGAAGTAGAATCCGTGAGGAATAGAAATG
OT3
gRNA site
PAM
WT 1 and 2
GTACAAACTCTCCTTTATAGTGAAACTCCTGGGGGAAAAGGGGGCT
C3
GTACAAACTCTCCTTTATAGTGAAACTCCTGGGGGAAAAGGGGGCT
G2
GTACAAACTCTCCTTTATAGTGAAACTCCTGGGGGAAAAGGGGGCT
D4
GTACAAACTCTCCTTTATAGTGAAACTCCTGGGGGAAAAGGGGGCT
I4
GTACAAACTCTCCTTTATAGTGAAACTCCTGGGGGAAAAGGGGGCT
OT4
PAM
gRNA site
WT 1 and 2
AACTTCCAAATACCACACGTGTTGCCCTACAAAGACCAAAAAGAGACAAA
C3
AACTTCCAAATACCACACGTGTTGCCCTACAAAGACCAAAAAGAGACAAA
G2
AACTTCCAAATACCACACGTGTTGCCCTACAAAGACCAAAAAGAGACAAA
D4
AACTTCCAAATACCACACGTGTTGCCCTACAAAGACCAAAAAGAGACAAA
I4
AACTTCCAAATACCACACGTGTTGCCCTACAAAGACCAAAAAGAGACAAA
OT5
gRNA site
PAM
WT 1 and 2
CTTTGTGAGCCGTGCTTTGAAGTGGAACACATCAGGGACGGCAGCARAAA
C3
CTTTGTGAGCCGTGCTTTGAAGTGGAACACATCAGGGACGGCAGCAGAAA
G2
CTTTGTGAGCCGTGCTTTGAAGTGGAACACATCAGGGACGGCAGCAGAAA
D4
CTTTGTGAGCCGTGCTTTGAAGTGGAACACATCAGGGACGGCAGCAGAAA
I4
CTTTGTGAGCCGTGCTTTGAAGTGGAACACATCAGGGACGGCAGCARAAA

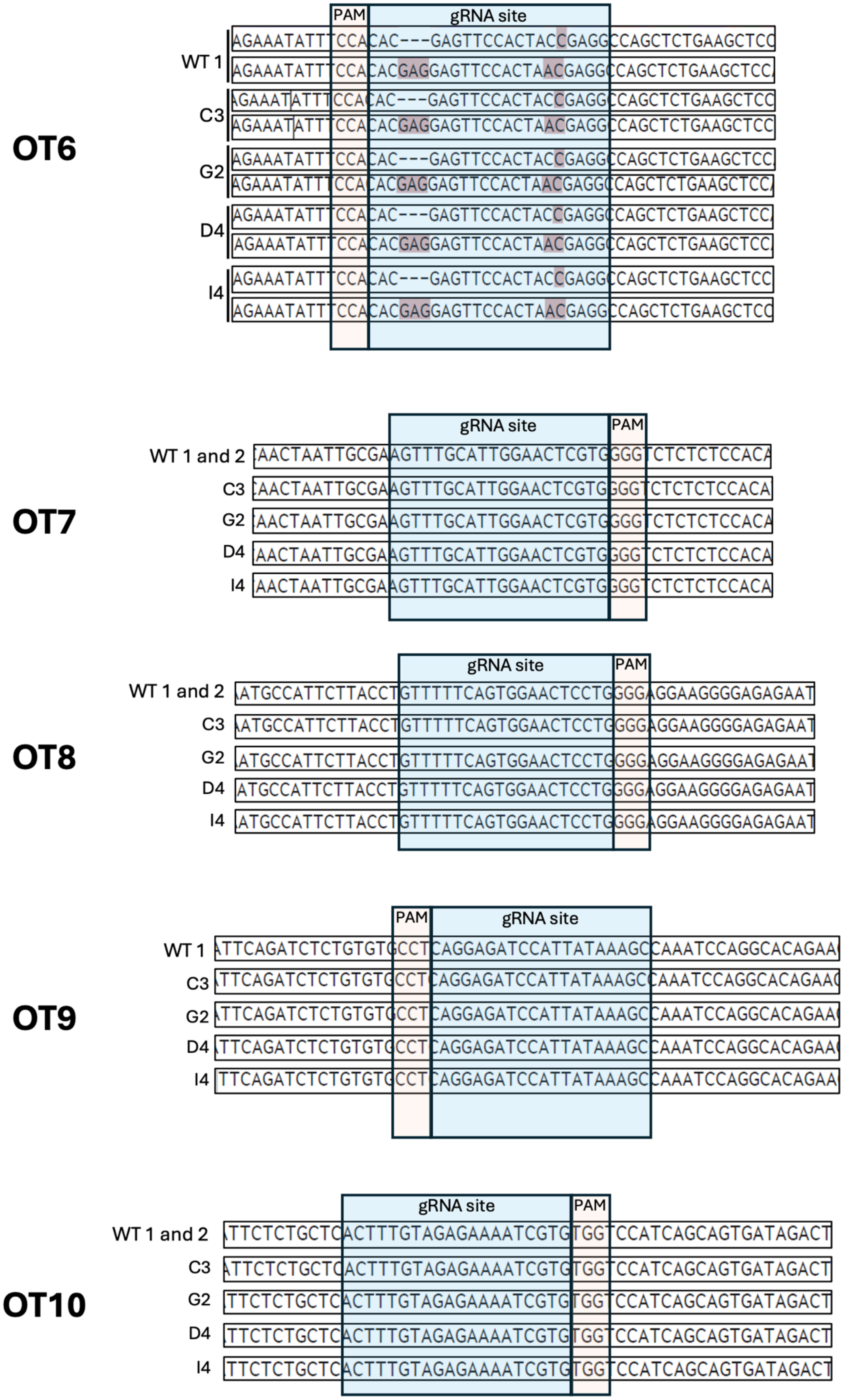
OT6
PAM
gRNA site
WT 1
C3
G2
D4
I4
OT7
WT 1 and 2
OT8
OT9
OT10

Figure S2. Characterization of potential off-target effects in the pig genome.

Alignment of sequencing results for the 10 potential off-target (OT) sites in four ABCA4 KO clones (#C3, G2, D4 and I4). Sequencing results from DNA from WT animals, either related (WT1) or unrelated (WT2) to the KO pig colony, processed in parallel, were included as reference. Letters highlighted in red represent mismatches compared to the NCBI GCF_000003025.6 reference genome. However, these mismatches were found also in untreated WT animals and were therefore considered unrelated to any genome editing effects.

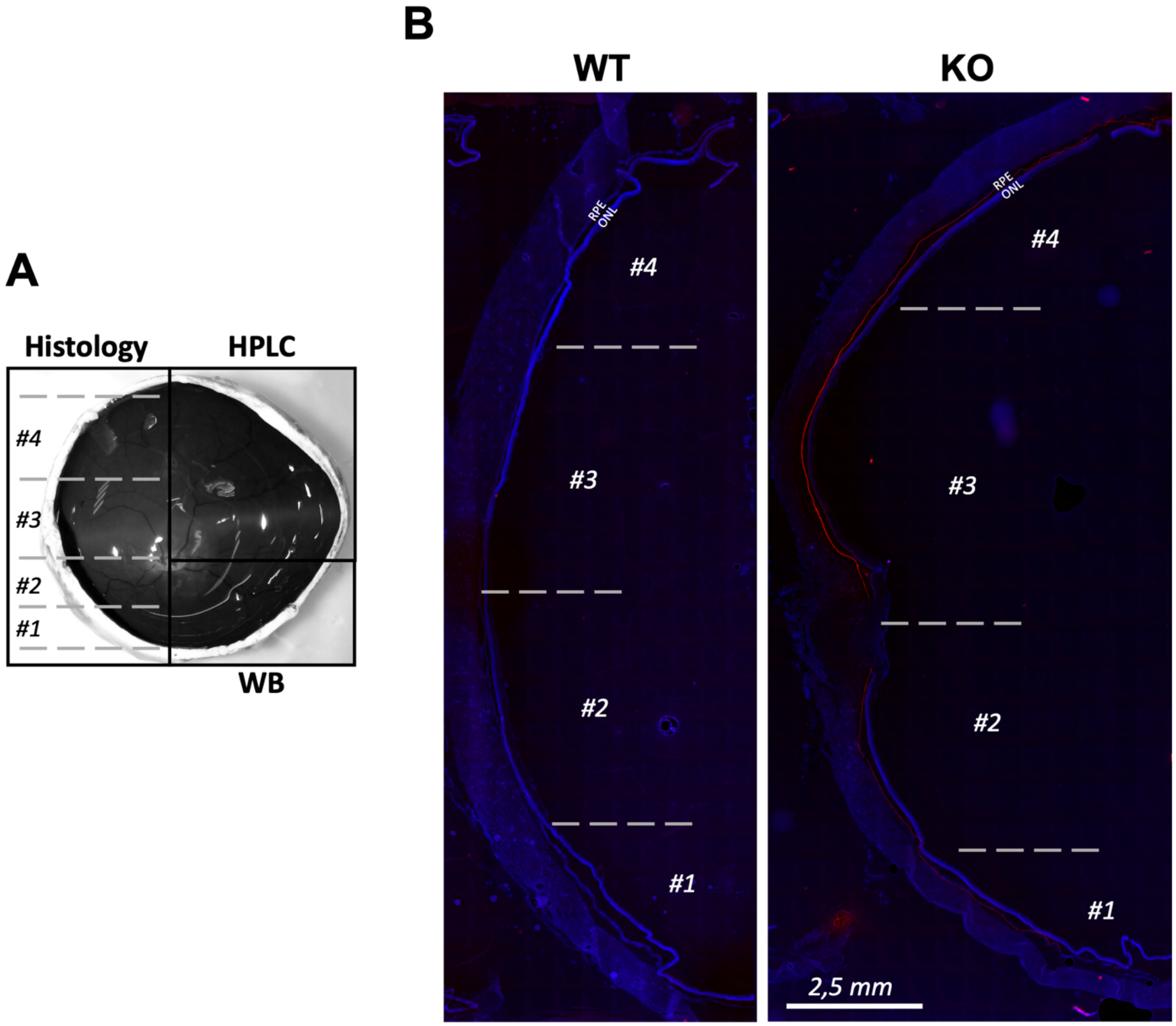

Figure S3. Pig eye harvest and analysis.

(A) Pig eyes were divided in 3 parts used for either: retina collection for Western Blot (WB) analysis of ABCA4 expression (lower right part); Retinal Pigmented Epithelium (RPE) collection for High Performance Liquid Chromatography (HPLC) quantification of A2E levels (upper right part); analysis of lipofuscin accumulation and retinal thickness on histological sections [virtually separating the retina in 4 retinal regions according to the cone density described in (6)].

(B) Representative images from retinal cryosections of wild-type (WT) and STGD1 (KO) pig eyes show extensive lipofuscin accumulation in the KO RPE (red autofluorescence), which is more evident in the area (#3) with high cone density. RPE: retinal pigment epithelium; ONL: outer nuclear layer.

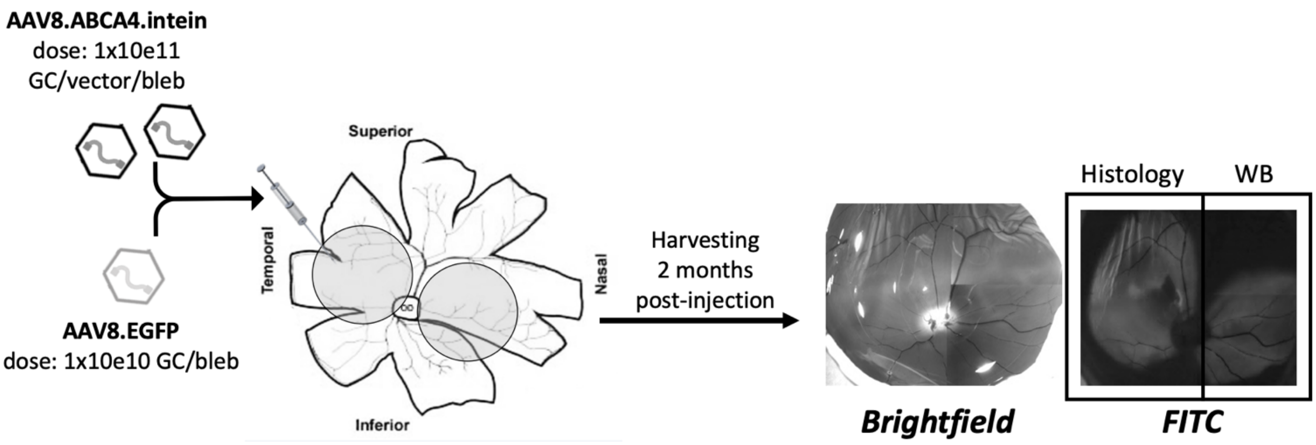

Figure S4. Schematic representation of STGD1 pig eyes subretinal injection with AAV8.*ABCA4*.intein vectors and harvesting.

At harvesting, STGD1 pig eyes were visualized under a stereomicroscope using the FITC filter to identify transduced (EGFP+) regions. Accordingly, eyes were divided in two pieces as follows: 1. the transduced area derived from blebs within the right half of the eyecup (either nasal or temporal region depending on whether right or left eyes were being dissected, respectively) were used for Western Blot analysis of ABCA4 protein expression; 2. the whole left portion of the eyecups (either temporal or nasal region depending on whether right or left eyes were being dissected, respectively) including transduced and untransduced areas derived from the blebs, were used for histological analysis to assess lipofuscin accumulation.

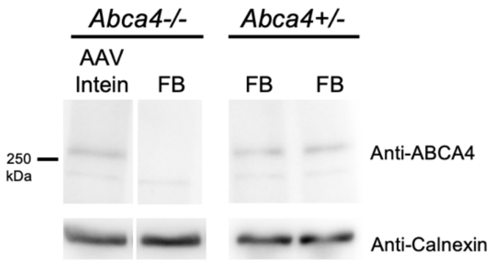

Figure S5. Subretinal injections in *Abca4-/-* mice of the AAV8.*ABCA4*.intein vectors produced for the NHP studies effectively reconstitute full-length ABCA4.

Western Blot analysis of *Abca4*-/- mouse retinal lysates 4 weeks post injection with AAV8.*ABCA4*.intein vectors at a dose of 4,5x10e9 GC of each virus/eye (n=2). *Abca4*+/- eyes injected with formulation buffer (FB; n=2) were used to indicate reference levels of expression of ABCA4 in normal retinas. Anti-calnexin is used as loading control.

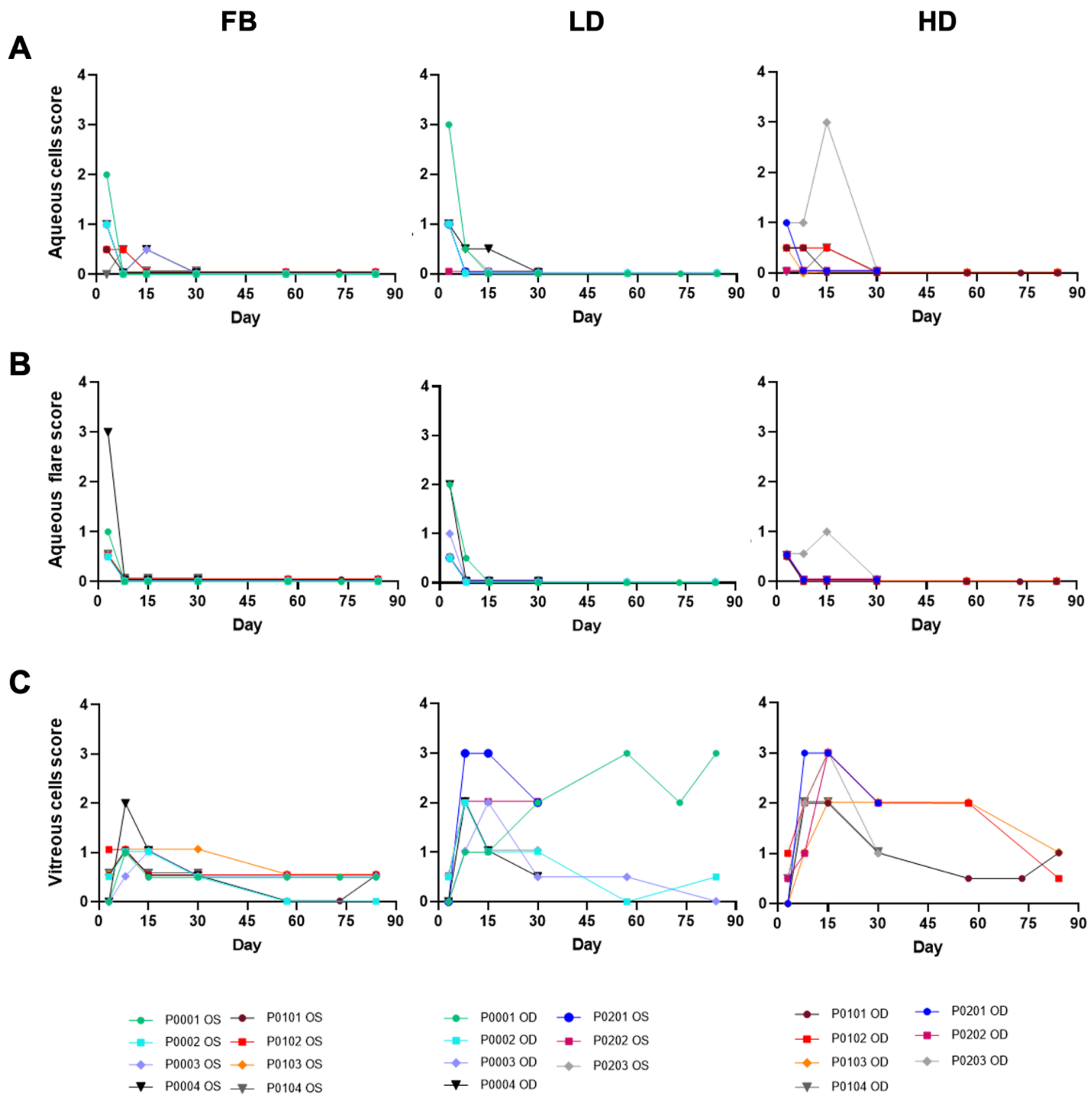

Figure S6. Intraocular inflammation in NHPs upon AAV8.*ABCA4*.intein vectors subretinal administration.

Aqueous cells (A) and flare (B) and vitreous cells (C) were monitored in injected eyes at different time points post-injection. A score (from 1 to 4) was attributed to define the severity of the ocular findings in each eye. Each colored line represents one eye. The ID of the eyes is indicated at the bottom of the figure. FB: eyes treated with the formulation buffer; LD: eyes treated with a low dose of AAV8.*ABCA4*.intein vectors; HD: eyes treated with a high dose of AAV8.*ABCA4*.intein vectors; OD: right eye; OS: left eye.

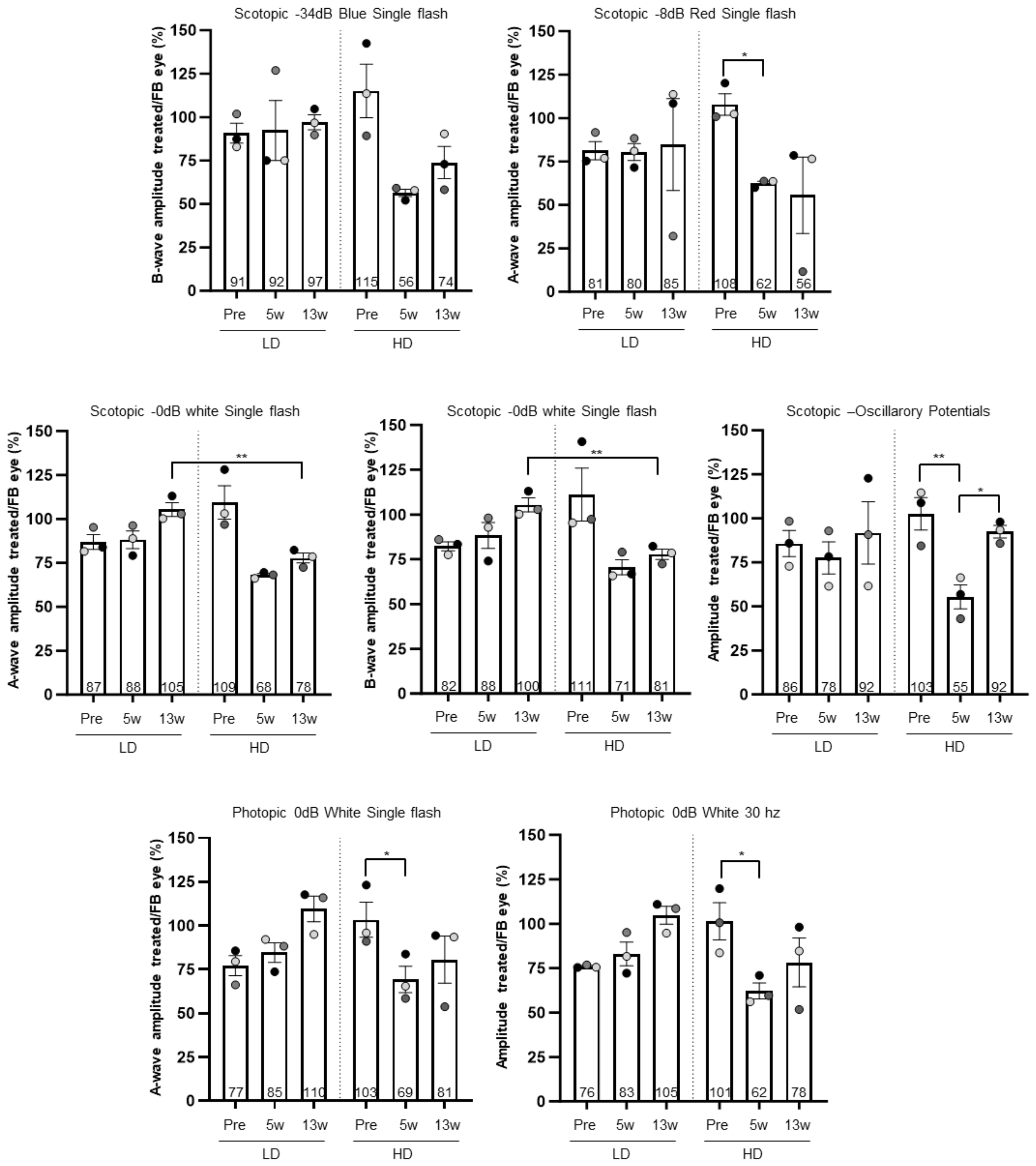

Figure S7. Electroretinogram responses in NHP eyes upon subretinal administration of AAV8.*ABCA4*.intein vectors.

ERG values in right eyes treated with either a low (LD) or a high (HD) dose of AAV8.*ABCA4*.intein vectors are expressed as percentage relative to left formulation buffer (FB)-treated eyes. Data are

presented as mean ± standard error of the mean. Each dot represents one eye. Pre: analysis performed before injection; w: week post-injection. Statistical comparisons were performed using the two-way ANOVA followed by Tukey's post hoc; * p value < 0.05; ** p value < 0.01. Further details on statistical analysis, including exact p values, can be found in the Table S7.

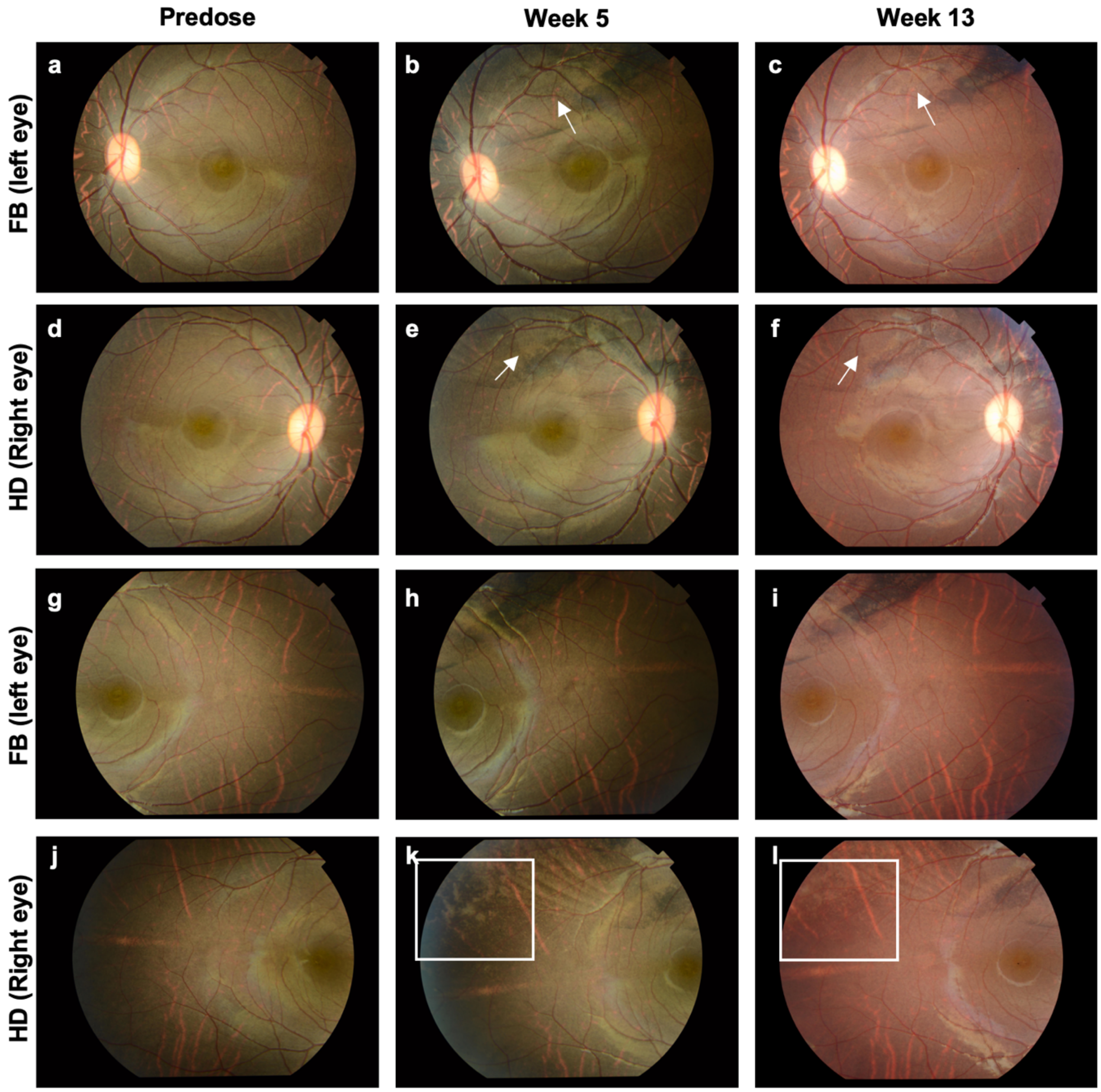

Figure S8. Fundus photography of NHP eyes upon subretinal administration of AAV8.*ABCA4*.intein vectors.

Fundus photography was performed in right eyes treated with AAV8.*ABCA4*.intein vectors at either a low dose (LD, n=3) or a high dose (HD, n=3) and in left eyes treated with the formulation buffer (FB, n=6). The figure shows representative fundus photographs of the: left eye before (A and G) and after (B,C,H,I) FB subretinal injection and right eye before (D and J) and after (E, F, K, L) subretinal injection of AAV8.*ABCA4*.intein vectors at HD. The white arrows indicate bleb-associated retinal pigmented epithelium (RPE) mottling; the white squares indicate RPE dappling at Week 5 or mottling at Week 13.

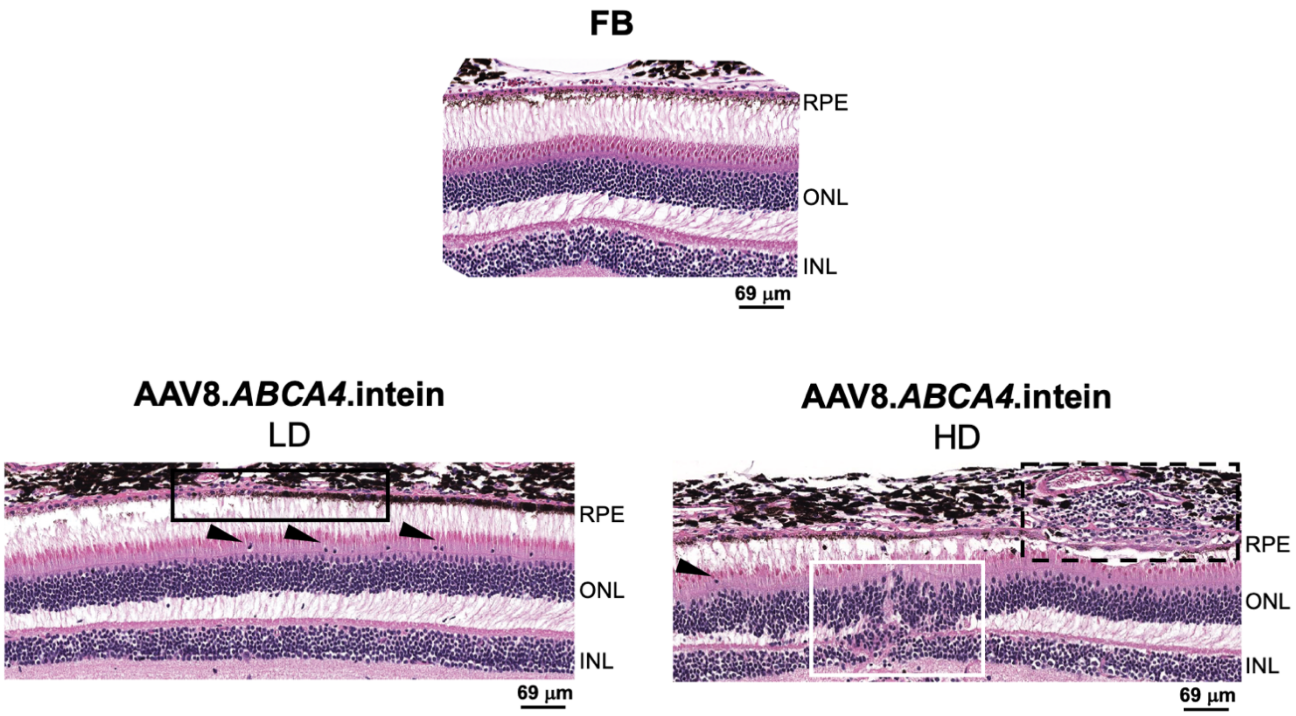

Figure S9. Minimal and focal RPE and retinal degeneration in eyes injected with AAV8.*ABCA4*.intein vectors.

Hematoxylin and eosin staining of retinal sections from NHPs eyes either injected with Formulation buffer (FB) or injected either with a low (LD) or a high (HD) dose of AAV8.*ABCA4*.intein vectors. Black arrowheads indicate displaced nuclei; the black box (in AAV8.*ABCA4*.intein LD) indicates areas at the interface between normal and hypertrophic/hyperpigmented RPE (on the right); the white box (in AAV8.*ABCA4*.intein HD) indicates areas of focal outer retina degeneration; the dashed black box (in AAV8.*ABCA4*.intein HD) indicates mononuclear cell infiltrates. RPE: retinal pigment epithelium; ONL: outer nuclear layer; INL: inner nuclear layer.

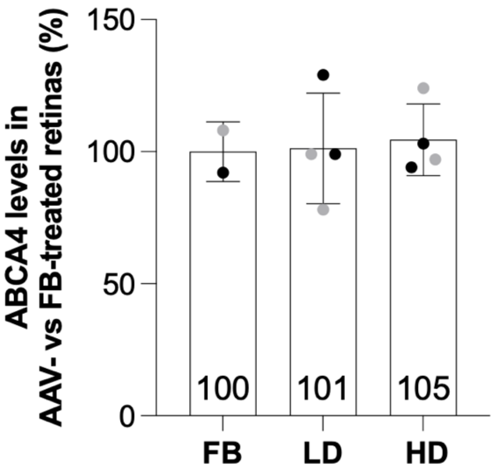

Figure S10. No differences in ABCA4 expression levels between AAV8.*ABCA4*.intein- and FB-treated retina samples from the not injected area

ABCA4 protein levels were measured by Simple Western analysis in two retina punches [one inferior temporal (black dot) and one inferior nasal (grey dot) punch] collected from the not-injected area of eyes receiving either a subretinal injection of AAV8.*ABCA4*.intein at the low (LD, n=2) or high (HD, n=2) dose or formulation buffer (FB, n=1), as control. ABCA4 protein levels were normalized to the endogenous control (PDE6B) and then expressed as percentage (%) of ABCA4 protein levels in AAV- vs FB-treated retinas. Columns represent mean values for group of treatment, while dots represent value of each retina punch. Data are reported as mean ± standard error of the mean.

Table S1. Top 10 off-target sites analysed in the STGD1 pig genome

Ref genome: NCBI GCF_000003025.6	***Ref genome: ensembl 76 ensSusScr***	***Off-target predicted score***
NC_010444.4_27693390	HIPK3-ENSSSCG00000030563	0.5882
NC_010456.5_27084120	ENSSSCG00000009753-ENSSSCG00000020440	0.4242
NC_010457.5_135267420	ENSSSCG00000025762-ENSSSCG00000024859	0.3174
NC_010443.5_129751748	ENSSSCG00000004743|TYRO3	0.2406
NC_010443.5_76434481	SLC22A16	0.2360
NC_010451.4_131781324	TRAF5	0.2310
NC_010460.4_37043354	ELMO1	0.2295
NC_010445.4_55041548	LYG2|ENSSSCG00000008183	0.1984
NC_010443.5_43350097	ENSSSCG00000030488|CEP85L	0.1632
NC_010449.5_102236416	ENSSSCG00000028718	0.1600

Table S2. Quality control tests on AAV8.*ABCA4.*intein test article, intermediates and formulation buffer/Vehicle.

Test	AAV8.5'h*ABCA4.N-intein* (Vector 1)			AAV8.C-intein.3'*hABCA4* (Vector 2)		
	Sub-lot #3738	Sub-lot #3739	Sub-lot #3740	Sub-lot #3741	Sub-lot #3742	Sub-lot #3743
**Gc titer (gc/ml)**	4.9×10^{12}	2.6×10^{12}	4.8×10^{12}	4.6×10^{12}	6.6×10^{12}	4.1×10^{12}
**Sterility**	No growth	No growth	No growth	No growth	No growth	No growth
**Purity (SDS-Page)**	Pass	Pass	Pass	Pass	Pass	Pass
**Endotoxin (EU/ml)**	0.243	0.219	0.208	0.227	0.312	0.209
	**Pooled AAV8.5'h*ABCA4.N-intein* (Vector 1)**			**Pooled AAV8.C-intein.3'*hABCA4* (Vector 2)**		
**Gc titer (gc/ml)**	3.7×10^{12}			4.3×10^{12}		
**Sterility**	No growth			No growth		
**AAV serotype Identity (WB)**	Pass			Pass		
**Full to empty (ELISA)**	49%			57%		
**Identity by sequencing**	Conform to the reference sequence			Conform to the reference sequence		
	**AAV8.*ABCA4.intein***					
**Gc titer (gc/ml)**	3.2×10^{12}					
**Infectious titer (TCID50, IU/ml)**	1.14×10^{10}					
**Sterility (EP 2.6.1-USP 71)**	No growth of bacteria and fungi					
**Endotoxin (EU/ml)**	0.169					
**Osmolality (mOsm/kg)**	353					
**pH**	7.1					
	**Formulation Buffer/Vehicle**					
**Sterility (EP 2.6.1-USP 71)**	No growth of bacteria and fungi					
**Endotoxin (EU/ml)**	0.05					
**Osmolality (mOsm/kg)**	352					
**pH**	7.25					

Sublots of either AAV8.5'h*ABCA4.N-intein* (Vector 1) or AAV8.C-intein.3'*hABCA4* (Vector 2) were pooled together to generate the pooled Vector 1 and pooled Vector 2 respectively. Then, 12.3 ml of pooled Vector 1 and 10,6 ml of pooled Vector 2 and 0.1 ml of Formulation Buffer were mixed and sterile filtered to generate the AAV8.*ABCA4.*intein lot used in NHPs. All tests were performed by Innovavector srl, with the exception of the following: identity by sequencing performed by Eurofins Genomics Europe Sequencing GMbh (Constance, Germany); infectious titer (TCID50) performed by Genosafe SAS (Evry, France) and sterility (EP2.6-1/USP 71) performed by SGS vitrology (Moredum Scientific; Glasgow, United Kingdom).

Table S3. Detailed study plan.

	Predose	Day 3	Day 8	Day 15	Week 5	Week 9	Week 13
**Opthalmic examinations/ Intraocular pressure**	X	X	X	X	X	X	X
**ERG/FP/OCT**	X				X	X (OCT)	X
**Ocular/Brain Histopathology[1]**					X		X
**BaseScope[2]**							X
**Simple Western[3]**					X		

Electroretinograms/Optical coherence tomography/Fundus Photography: n eyes=3 for each group; Ophthalmic examinations/Intraocular pressure: all animals available.

[1]Histopathology: week 5, n eyes=2 for LD and HD, n eyes=1 for FB; week 13 n eyes=3 for each group;

[2]BaseScope: n eyes=3 for each group.

[3]Simple Western: n eyes =2 for LD and HD, n eyes=1 for FB.

FB: eyes treated with the formulation buffer; LD: eyes treated with a low dose of AAV8.*ABCA4*.intein vectors; HD: eyes treated with a high dose of AAV8.*ABCA4*.intein vectors; ERG: electroretinograms; FP: fundus photography; OCT: optical coherence tomography.

Table S4. Intraocular pressure.

	Intraocular pressure (mm Hg)		
**Timepoint**	**FB**	**LD**	**HD**
**Predose**	18.1 ± 1.3	15.1 ± 0.7	19.4 ± 1.1
**Day 3**	10.3 ± 1.8	12.9 ± 1.7	11.3 ± 1.4
**Day 8**	17.3 ± 1.3	14.9 ± 1.1	17.1 ± 0.8
**Day 15**	14.4 ± 1.2	12.4 ± 0.9	14.0 ± 1.1
**Day 30**	15.9 ± 0.9	15.3 ± 1.5	17.6 ± 0.8
**Day 57**	14.8 ± 0.8	13.3 ± 2.3	17.0 ± 2.1
**Day 85**	16.7 ± 1.4	13.0 ± 0.6	18.0 ± 0.0

Data are reported as mean ± standard error of the mean.

FB: eyes treated with AAV formulation buffer.

LD: eyes treated with AAV8.*ABCA4*.intein at the low dose.

HD: eyes treated with AAV8.*ABCA4*.intein at the high dose.

The number of eyes is as follows: FB, n=8 except for Day 57 and 85, n=6; LD and HD, n=7, except for Day 57 and 85, n=3.

Table S5. Incidence and severity of OCT findings

1. **Findings with higher incidence in AAV8.*ABCA4*.intein-injected eyes relative to formulation buffer (FB)**

		FB (n=6)	LD (n=3)	HD (n=3)
**Patches of increased HRM/SHRM**	Predose	0/6	0/3	0/3
	Week 5	1/6	2/3	1/3
	Week 9	1/6	1/3	2/3
	Week 13	1/6	1/3	1/3
**Areas with thinned photoreceptors layers with HRF**	Predose	0/6	0/3	0/3
	Week 5	2/6	3/3	3/3
	Week 9	2/6	3/3	3/3
	Week 13	1/6	3/3	3/3

2. **Findings with higher, dose-dependent incidence in AAV8.*ABCA4*.intein-injected eyes relative to formulation buffer (FB)**

		FB (n=6)	LD (n=3)	HD (n=3)
**Patches of moderate EZ, IZ, RPE disorganization**	Predose	0/6	0/3	0/3
	Week 5	0/6	0/3	1/3
	Week 9	0/6	0/3	1/3
	Week 13	0/6	2/3	3/3
**Areas of ONL thinning**	Predose	0/6	0/3	0/3
	Week 5	0/6	0/3	0/3
	Week 9	0/6	0/3	0/3
	Week 13	0/6	0/3	2/3

HRM: Hyperreflective Material.

SHRM: Subretinal Hyperreflective Material.

HRF: Hyperreflective Foci.

EZ: Ellipsoid zone.

IZ: Interdigitation zone.

RPE: retinal pigment epithelium.

ONL: Outer Nuclear Layer.

FB: eyes treated with AAV formulation buffer.

LD: eyes treated with AAV8.*ABCA4*.intein at the low dose.

HD: eyes treated with AAV8.*ABCA4*.intein at the high dose.

Table S6. Primers used for amplification of off-target sites

	FW	RV
**OT 1**	CCTGACTCCAGATGGTAAGT	CTAGAGGATGTTGTGAGGTCC
**OT 2**	GCAGAACAAAACTAGAGGGG	GCAGGTACCAACTGAGATCCT
**OT 3**	TCAGCACATGGGCTAAGATA	CAGTGAAGACAAAGTCCAGGA
**OT 4**	AAGGGGAGAACAGCTATGAA	AACTGGGTAAGAGGTACACAG
**OT 5**	GTGACACTACCTGCTCTAGT	CTGAAGTGTCTCCTCCCAAAT
**OT 6**	TTAAACAAGCACAGAGGACG	ATGACATCCTGACCATACTCC
**OT 7**	CAGGATTTCTGAGCTAGGGA	TCTGGACTGTTTACCACCATG
**OT 8**	TCTGAAGCCGCATGTTTAAA	TCCTGTGACATAAGGGAATGC
**OT 9**	AGTGTCACATAGGTAGCCAA	CTCACCTAGTCCCAACTGTTT
**OT 10**	CCACATATACGGTGCTTCTG	TTTCATTGGCAGCTGTCTTAC

OT: off-target site

Table S7. Details of the statistical analysis on ERG data from NHP studies

	2-way Anova (*p* value)				Multiple comparisons -Tukey post-hoc test (*p* value)								
**Endpoint**	**Dose x Timepoint**	**Dose**	**Timepoint**	**Subject**	**LD**			**HD**			**LD vs HD**		
					w5 vs Pre	*w13 vs Pre*	*w5 vs w13*	*w5 vs Pre*	*w13 vs Pre*	*w5 vs w13*	*Pre*	*w5*	*w13*
Scotopic -34dB Blue Single flash - Bwave	**0,0432**	0,2762	0,0737	0,3823	0,9899	0,7956	0,9729	0,1386	0,1797	0,3632	0,2491	0,1710	0,1143
Scotopic -8dB Red Single flash – A wave	0,2229	0,5407	0,2762	0,6320	0,9322	0,9934	0,9886	**0,0467**	0,1952	0,9519	0,0323	0,0587	0,4445
Scotopic -8dB Red Single flash – B wave	**0,0076**	0,6220	**0,0147**	0,2305	0,3817	0,3343	**0,0360**	0,1216	0,0649	0,3231	0,1799	**0,0170**	**0,0414**
Scotopic -0dB white Single flash– A wave	**0,0013**	0,1594	**0,0074**	0,2517	0,9347	0,1704	0,3028	0,0777	0,0849	0,1258	0,1282	0,0506	**0,0062**
Scotopic -0dB white Single flash– B wave	**0,0128**	0,3684	0,1426	0,5730	0,7486	**0,0449**	0,4320	0,2409	0,2099	0,6251	0,1874	0,1196	**0,0062**
Scotopic -0dB white Oscillatory Potentials	0,0568	0,9019	**0,0060**	**0,0343**	0,6843	0,8079	0,3491	**0,0029**	0,5614	**0,0118**	0,2532	0,1413	0,9629
Photopic -0dB white Single flash– A wave	**0,0089**	0,5567	0,0804	0,0904	0,7674	0,1459	0,2967	**0,0112**	0,2785	0,5598	0,1024	0,1818	0,1509
Photopic -0dB white Single flash– B wave	**0,0089**	0,3910	0,1061	0,4417	0,3560	0,1707	0,2966	0,0760	0,2483	0,6987	0,0825	**0,0403**	0,1091
Photopic -0dB white 30 hz– B wave	**0,0081**	0,4353	0,0627	0,1301	0,5854	0,0511	0,2172	**0,0437**	0,4129	0,5024	0,1346	0,0691	0,1839

LD: eyes treated with a low dose of AAV8.*ABCA4*.Intein vectors.
HD: eyes treated with a high dose of AAV8.*ABCA4*.Intein vectors.
Pre: analysis performed before injection; w: week post-injection.
